# The Somatosensory Mismatch Negativity

**DOI:** 10.1111/ejn.70301

**Published:** 2025-11-25

**Authors:** Miro Grundei, Wolfger von der Behrens, Piia Astikainen, Nicole Wetzel, Felix Blankenburg

**Affiliations:** ^1^ Neurocomputation and Neuroimaging Unit Freie Universität Berlin Berlin Germany; ^2^ Institute of Neuroinformatics University and ETH Zurich Zurich Switzerland; ^3^ Department of Psychology University of Jyväskylä Jyväskylä Finland; ^4^ Leibnitz Institute for Neurobiology Magdeburg Germany

**Keywords:** ERP, mismatch negativity, predictive processing, somatosensory system, surprise

## Abstract

Mismatch negativity (MMN) is a well‐established neural signature of automatic change detection in the auditory modality. Growing evidence suggests that analogous responses exist in other sensory domains, including somatosensation. This review provides an initial overview of the somatosensory MMN (sMMN), summarizing findings from both human and animal research concerning its underlying neural mechanisms and functional significance as a predictive error signal. We discuss electrophysiological and neuroimaging evidence identifying primary and secondary somatosensory cortices (S1 and S2) as key generators of the sMMN, highlighting the roles of sensory and stimulus‐specific adaptation, as well as true deviance detection or surprise. Computational studies further support a hierarchical inference process, where somatosensory mismatch responses encode probabilistic structures based on transition probabilities of sequential input. We also address the clinical relevance of the sMMN in neurodevelopmental, psychiatric, and neurological disorders, as well as its role in aging and body representation. Understanding the neuronal mechanisms underlying the sMMN not only advances our knowledge of somatosensory predictive processing but also contributes to the broader study of perception and learning in the brain.

## Graphical abstract

The somatosensory mismatch negativity (sMMN) is a neural signature of automatic deviance detection, reflecting predictive processing in touch. This review synthesizes human, animal, and computational research to identify the key generators of the sMMN and examine its relation to adaptation, surprise, and probabilistic inference. Highlighting its clinical relevance for neurodevelopmental, psychiatric, and neurological conditions, we explore how the sMMN provides valuable insights into perception, learning, and aging in the somatosensory system.

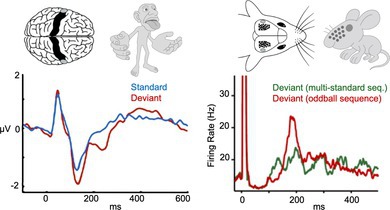

**Abbreviations**
BS: Bayesian surprise
DC: Dirichlet categorical (model)
ECoG: Electrocorticography
EEG: Electroencephalography
ERP: Event‐related potential
HMM: Hidden Markov model
L2/3: Layer 2/3
L4: Layer 4
L6: Layer 6
LFP: Local field potential
MEG: Magnetoencephalography
MMN: Mismatch negativity
MMR: Mismatch response
Ms: Milliseconds
MUA: Multi‐unit activity
N1: Negative auditory ERP component at around 100 ms
N140: Negative somatosensory ERP component at around 140 ms
NMDA: N‐Methyl‐D‐aspartate
NMDAR: N‐methyl‐D‐aspartate receptor
P3: Positive ERP component at around 300 ms
P100: Positive somatosensory ERP component at around 100 ms
Pom: Posterior medial nucleus of the thalamus
PrV: Principal nucleus of the trigeminal nerve in the brainstem
RF: Receptive field
RA: Rapidly adapting (afferents)
S1: Primary somatosensory cortex
S2: Secondary somatosensory cortex
SA: Slowly adapting (afferents)
SSA: Stimulus‐specific adaptation
sMMN: Somatosensory mismatch negativity
SpVi: The interpolaris subnucleus of the spinal trigeminal nucleus in the brainstem
VPM: Ventral posterior medial nucleus of the thalamus

## 1 Introduction

The mismatch negativity (MMN), first described by Näätänen and colleagues in the auditory modality (Näätänen et al. 1978), is arguably the most well‐known electroencephalography (EEG) component reflecting the brain's response to violations of sensory regularities. Research on MMN has significantly influenced various fields of neuroscience, including perception, memory, development, and clinical psychiatry (see Näätänen et al. (2019) for review). More recently, MMN has been associated with the Bayesian brain hypothesis (Dayan and Abbott 2001; Knill and Pouget 2004) and the theory of predictive coding (Friston 2005), according to which the brain continuously learns statistical dependencies in the surrounding stimulus environment and makes predictions about future sensory events. In this context, MMN has been suggested to reflect a neuronal manifestation of prediction error if sensory input diverges from internal predictions (Friston 2005; Garrido, Kilner, Stephan, and Friston 2009).

Last year marked the 10th edition of the MMN conference, dedicated to advancing the understanding of the properties and applications of MMN and related neuronal responses. Although most research pertains to the auditory modality, an increasing number of studies report findings on comparable responses to MMN in other sensory modalities, such as somatosensation and vision (for a recent review on the visual MMN, see Czigler and Kojouharova (2021)). Investigating this neuronal signature across different senses is crucial for distinguishing modality‐specific from cross‐modal properties, advancing a comprehensive mechanistic understanding of the response and potentially offering clinical benefits for special populations. As highlighted during the symposium “Somatosensory MMN, Prediction Error and Surprise,” somatosensory MMN (sMMN) holds significant potential for enhancing our understanding of the predictive nature of sensory processing. Therefore, we, the speakers at this symposium, decided to write a review article that provides an initial overview of the literature on sMMN. We review the existing evidence from both human and animal research regarding the presence of a neuronal signature akin to the auditory MMN in the somatosensory system, examine proposed underlying mechanisms, and evaluate its potential for investigating the brain's perceptual model of the sensory environment.

**FIGURE 1:**
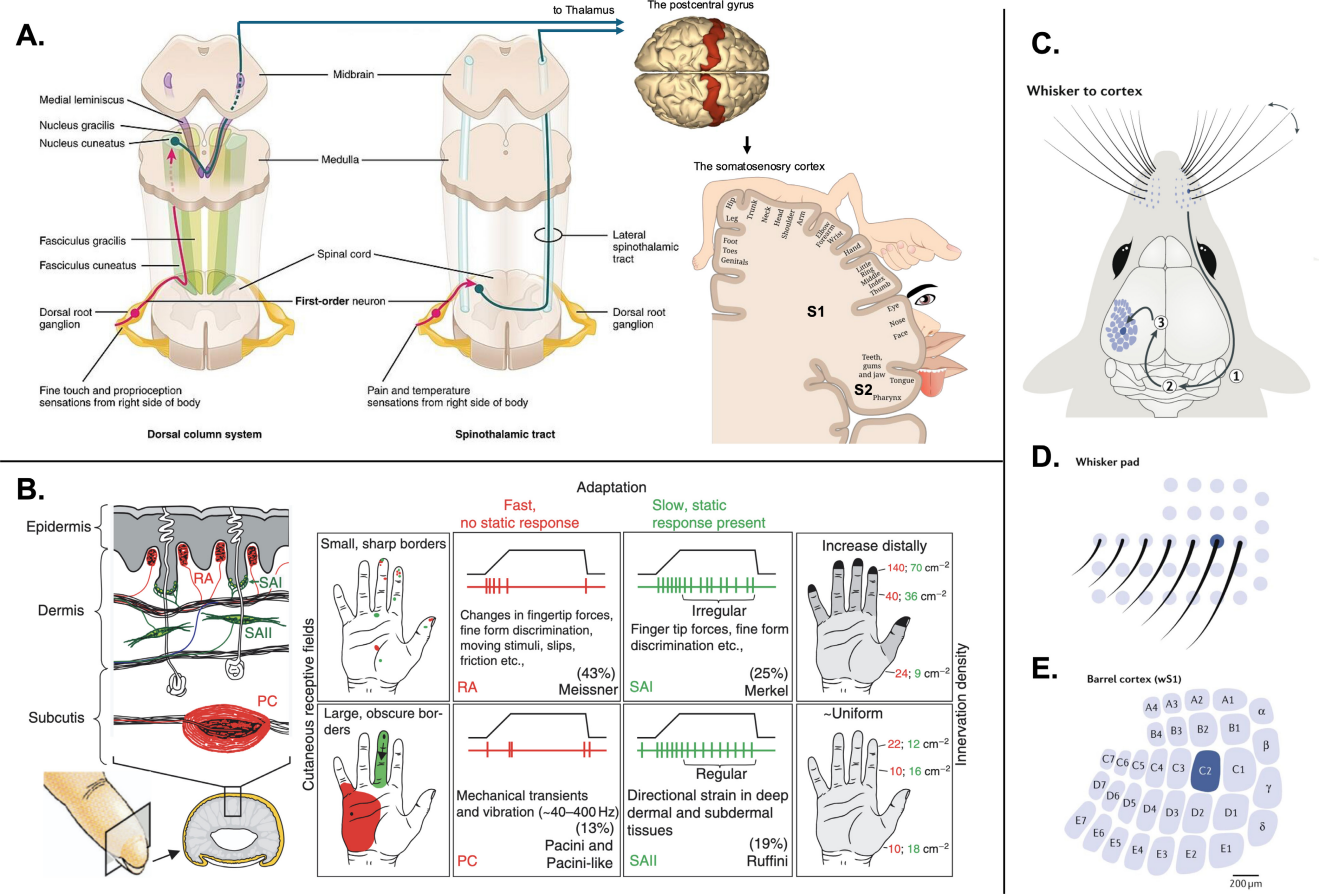
The somatosensory system. (A) The human somatosensory system. Left: Dorsal column pathway. Middle: Spinothalamic pathway. Right: The somatosensory cortex in postcentral gyrus with its somatotopic organization and the somatosensory homunculus of S1. *Adapted from Wikimedia Commons with permission (CC BY 3.0 and CC attribution‐share alike 2.1 Japan)*. (B) Cutaneous afferents in human glabrous skin (finger). Left: Schematic overview of major mechanoreceptor types and their distribution within the skin layers. Right: Functional properties of the four classes of cutaneous afferents, showing their adaptation behavior and receptive field (RF) sizes. Rapidly adapting afferents respond to transient indentation, whereas slowly adapting afferents encode sustained stimuli. Type I afferents are characterized by small, densely innervated RFs, while type II afferents exhibit larger RFs with lower innervation density. As an example, Pacinian corpuscle (PC) afferents belong to the rapidly adapting type II class, combining fast adaptation with large RFs and sparse innervation. *Reproduced from* Johansson and Flanagan (2008)*, with permission of Elsevier through PLSclear*. (C) Deflecting a mystacial whisker initiates sequential activation along the somatosensory pathway: first in trigeminal ganglion neurons (1), then in brainstem relay neurons (2), and subsequently in thalamic neurons (3), before signals reach the whisker‐related primary somatosensory cortex (S1). (D,E) In rodents (rats and mice), the S1 whisker region contains distinct anatomical structures known as “barrels.” Each barrel corresponds to a single mystacial whisker and together they form a precise somatotopic map. *Adapted from* Petersen (2019) *with permission*.

The somatosensory system encodes signals from the skin, muscles, and joints, supporting the perception of touch, vibration, texture, shape, and limb position (proprioception). In the skin, four classes of tactile mechanoreceptors provide distinct streams of input (Figure 1B; Delhaye et al. 2018). Slowly adapting type 1 (SA1) afferents, associated with Merkel cells, have small receptive fields with well‐defined borders and signal sustained pressure and edges with high spatial resolution. Slowly adapting type 2 (SA2) afferents, associated with Ruffini endings, have large receptive fields with poorly defined borders and respond to skin stretch, providing information about finger posture and proprioceptive cues. Rapidly adapting type 1 (RA1) afferents, linked to Meissner corpuscles, also have small, well‐defined receptive fields and detect transient deformation such as flutter and low‐frequency motion across the skin. Rapidly adapting type 2 (RA2) afferents, associated with Pacinian corpuscles, have very large receptive fields with poorly defined borders and are sensitive to high‐frequency vibrations, supporting fine texture perception through vibratory cues. These cutaneous inputs, together with proprioceptive signals from muscle spindles and joint receptors, ascend through the dorsal column–medial lemniscal pathway and converge in the primary somatosensory cortex (S1). S1 is organized somatotopically into four subregions (Brodmann Areas 3b, 3a, 1, and 2), which together form the classic “homunculus” representation of the body surface (Figure 1A; Delhaye et al. 2018; Penfield and Boldrey 1937; Kaas 1993). Each subregion makes a distinct contribution to tactile coding (Friedman et al. 2004). Area 3b is the primary cortical recipient of cutaneous input (also called “S1 proper”) and represents local skin indentation, vibrations, and spatially precise touch. Area 3a instead mainly encodes proprioceptive signals from muscles and joints, establishing a cortical map of limb position. Area 1 refines cutaneous input, emphasizing texture and vibration; here, modality‐specific signals from SA and RA afferents remain partially segregated in cortical “patches” at submillimeter scales, but are also integrated to support the perception of surface texture and fine tactile patterns (Friedman et al. 2004). Area 2 combines cutaneous and proprioceptive information, enabling representations of object shape, stereognosis, and tactile motion trajectories.

In rodents, S1 is also organized somatotopically, but with a distinctive posteromedial subfield that represents the whiskers. Each whisker on the contralateral snout maps onto a discrete neuronal cluster or “barrel,” creating a unique one‐to‐one correspondence between peripheral sensors and cortical modules (Woolsey and Van der Loos 1970; Petersen 2007). Surrounding the barrel field, additional subregions of S1 represent the limbs, trunk, tail, and oral structures in a continuous somatotopic arrangement, though these regions lack the sharply defined barrels of the whisker cortex (Waters et al. 1995). Analogous to primates, rodent S1 is subdivided into homologous regions that emphasize cutaneous versus proprioceptive input, with integration of these signals supporting representations of whisker deflection, body position, and object contact.

Beyond the initial processing in S1, tactile signals are relayed to the secondary somatosensory cortex (S2), which lies in the parietal operculum in (human and non‐human) primates and lateral to S1 in rodents. In both, S2 receives dense feedforward input from all subdivisions of S1 and sends reciprocal feedback projections. Compared to S1, receptive fields in S2 are larger, often spanning multiple digits or multiple whiskers, and neurons show greater sensitivity to higher‐order stimulus features. In primates, S2 encodes object curvature, surface motion, bilateral somatosensory integration, and other abstract tactile patterns (Delhaye et al. 2018; Iwamura 1998). In rodents, S2 integrates across whiskers and body parts to represent texture, direction of whisker motion, and bilateral input (Petersen 2007; Carvell and Simons 1990). Therefore, despite differences in cortical microstructure, the role of S2 as a higher‐order hub for integrating and transforming somatosensory input is conserved across species. Together, S1 and S2 form the initial stages of cortical tactile processing, transforming mechanoreceptor input into increasingly complex representations of the external world.

### 1.1 Somatosensory MMN—Electrophysiological Signature and Underlying Generators

**FIGURE 2:**
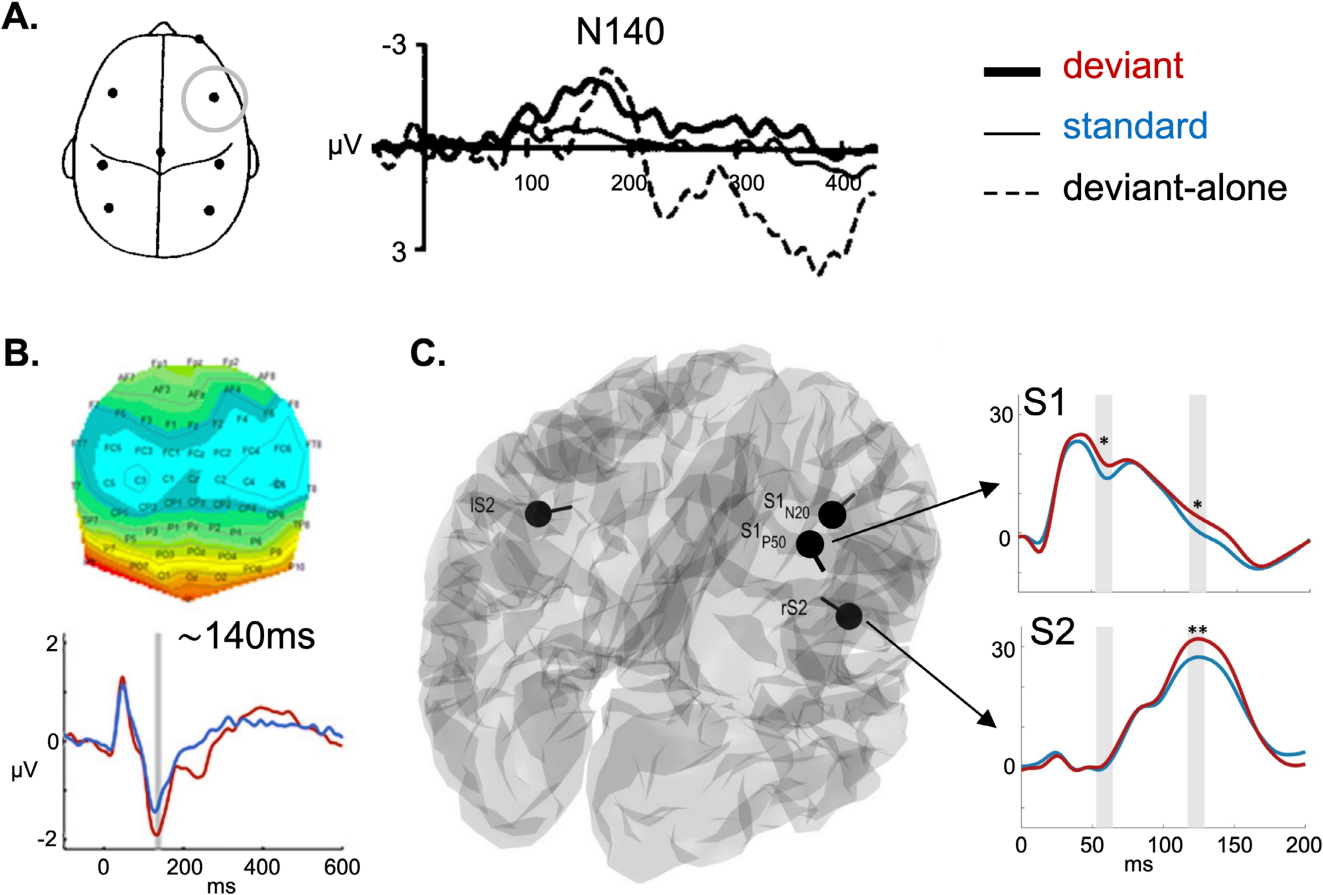
Somatosensory MMN. (A) One of the first reports of sMMN as an increased N140 response with shortened latency to deviants (thick black line) compared to standards (thin line) and deviant alone (thin dashed line). *Adapted from* Kekoni et al. (1997) *with permission*. (B) EEG topography of bilateral sMMN to roving‐oddball stimuli at 140 ms and increased deviant negativity at electrode C6. *Adapted from* Ostwald et al. (2012) *with permission*. (C) Somatosensory source model and trajectories of S1 and S2 activity with increased deviant responses to roving‐oddball stimuli at early (60 ms, S1) and mid‐latency time‐windows (130 ms, S1 and S2). *Adapted from* Gijsen et al. (2021) *with permission (CC BY 4.0)*.

In line with the multifaceted nature of somatosensory processing, diverse stimulation techniques have been employed to explore how the brain identifies deviant sensory events. First evidence for an sMMN was reported by Kekoni et al. (1997) (Figure 2), using a passive oddball paradigm, in which a repeated stream of identical vibrotactile (standard) stimuli to the fingers was occasionally interrupted by deviating stimuli. By comparing responses to deviants embedded within the standard stream versus the same stimuli presented in isolation (deviant‐alone condition), they found that location deviants (different finger) elicited a negativity between 100 and 200 ms, while frequency deviants elicited a negativity around 250 ms. These findings were interpreted as demonstrations of somatosensory MMN. The latency differences could potentially reflect contributions from distinct cortical sources: early location‐related responses arise in areas 3b and 1, which encode spatially precise cutaneous input, whereas later frequency‐related responses likely involve areas 1 and S2 where vibration and texture are processed.

In line with these initial findings, sMMN has since been elicited by deviance across multiple stimulus features, including location (Kekoni et al. 1997; Shinozaki et al. 1998; Akatsuka et al. 2005; Restuccia et al. 2007; Restuccia et al. 2009; Chen et al. 2008; Hu et al. 2013; Strömmer et al. 2014; Naeije et al. 2016; Naeije et al. 2018; Fardo et al. 2017; Shen et al. 2018a; Shen et al. 2018b; Hautasaari et al. 2019; He et al. 2020; Xu et al. 2021) (engaging spatial maps in areas 3b and 1), frequency (Kekoni et al. 1997; Spackman et al. 2007; Butler et al. 2012) (linked to vibration and texture processing in area 1 and S2), duration (Akatsuka et al. 2005; Spackman et al. 2007; Isenstein, Freedman, Xu, et al. 2024; Isenstein, Freedman, Molholm, and Foxe 2024; Isenstein et al. 2025; Akatsuka, Wasaka, Nakata, et al. 2007; Akatsuka, Wasaka, Nakata, Kida, and Kakigi 2007; Spackman et al. 2010) (involving temporal integration across S1 and S2), and intensity (Ostwald et al. 2012; Zhang et al. 2019; Gijsen et al. 2021; Grundei, Schröder, et al. 2023) (likely reflecting both early encoding in area 3b and integrative processing in S2). To deliver these manipulations, studies have utilized a range of precisely controlled tactile actuators, including vibrotactile stimulators (Kekoni et al. 1997; Spackman et al. 2007; Butler et al. 2012; Isenstein, Freedman, Xu, et al. 2024; Isenstein, Freedman, Molholm, and Foxe 2024; Spackman et al. 2010; Chen et al. 2014), pneumatic devices (Naeije et al. 2016; Naeije et al. 2018; Hautasaari et al. 2019; Zhang et al. 2019), inflatable membranes (Shen et al. 2018a; Shen et al. 2018b; Shen et al. 2020; Andersen and Lundqvist 2019; Dercksen et al. 2024), and brief electrical pulses to peripheral nerves (Chen et al. 2008; Ostwald et al. 2012; Gijsen et al. 2021; Grundei, Schröder, et al. 2023; Huang et al. 2005; Duerler et al. 2021; Grundei, Schmidt, and Blankenburg 2023), to the hand (Akatsuka et al. 2005; Hu et al. 2013; Akatsuka, Wasaka, Nakata, et al. 2007; Akatsuka, Wasaka, Nakata, Kida, and Kakigi 2007) or the fingers (Shinozaki et al. 1998; Restuccia et al. 2007; Strömmer et al. 2014; Hautasaari et al. 2019; Xu et al. 2021). These approaches afford fine‐grained control over stimulus properties. However, as described above, different stimulation methods engage distinct mechanoreceptor classes and afferent pathways (Figure 1), influencing both peripheral encoding and the cortical feature representations that emerge across S1 and S2.

Differences in experimental procedures shape the neuronal dipoles that underpin the sMMN and likely contribute to the considerable inconsistencies observed in reported EEG scalp distributions. While some studies converge on sensor‐level responses contralateral to the stimulation site, with dominance of temporal (Hu et al. 2013; Strömmer et al. 2014; Shen et al. 2018b; Zhang et al. 2019; Gijsen et al. 2021; Grundei, Schröder, et al. 2023) or parietal (Restuccia et al. 2007) electrode sites, other studies also report frontal and fronto‐central (Kekoni et al. 1997; Shinozaki et al. 1998; Akatsuka et al. 2005; Restuccia et al. 2009; Fardo et al. 2017; He et al. 2020; Isenstein, Freedman, Xu, et al. 2024; Isenstein, Freedman, Molholm, and Foxe 2024; Isenstein et al. 2025) or central (Shen et al. 2018a; Spackman et al. 2010; Ostwald et al. 2012) effects. This variability contrasts with the auditory MMN, which shows a consistent fronto‐central distribution. The discrepancy raises two possibilities: that tactile mismatch responses recruit more heterogeneous neural networks, and that different stimulation procedures may engage somatosensory areas in distinct ways. Supporting the latter idea, Hautasaari and colleagues demonstrated diverging recruitment of somatosensory cortex during mismatch responses (MMRs) elicited in magnetoencephalography (MEG) by tactile versus electrical stimulation (Hautasaari et al. 2019). Note that we refer to the term MMR to denote any measured difference between standard and deviant stimuli—regardless of polarity, modality, or method (e.g., EEG, MEG, intracranial electrophysiology)—in contrast to MMN. The source reconstruction results revealed a more sustained involvement of S1 in response to tactile stimulation during early (~50 ms) and midlatency (~150 ms) MMRs, whereas both tactile and electrical stimulation recruited S2 around ~150 ms. The additional sustained and bilateral S1 involvement with tactile deviants may relate to differences at the receptor level: tactile stimulation selectively activates cutaneous mechanoreceptors and Aβ afferents, producing temporally precise and spatially specific input. Such coherent input not only drives the early contralateral S1 response but can also be integrated across hemispheres at later stages. In contrast, electrical stimulation excites a heterogeneous mix of fibers with varying conduction velocities, potentially generating less selective afferent and more restricted cortical engagement. Given the relatively small number of studies in the somatosensory domain, current evidence does not yet support a consistent mapping between stimulation procedures and specific sMMN features, underscoring the need for future work to systematically investigate these factors.

Although there is also some variability in the reported latencies, the sMMN occurs primarily between 100 and 250 ms post‐stimulus onset, which has been related to an increased somatosensory N140 in EEG (Kekoni et al. 1997). Additionally, however, a considerable number of studies have reported an early component between 50 and 100 ms reflecting mismatch between standard and deviant stimuli (Shinozaki et al. 1998; Akatsuka et al. 2005; Naeije et al. 2016; Xu et al. 2021; Akatsuka, Wasaka, Nakata, et al. 2007; Akatsuka, Wasaka, Nakata, Kida, and Kakigi 2007; Ostwald et al. 2012; Gijsen et al. 2021; Grundei, Schröder, et al. 2023). Deviant processing reflected in early and mid‐latency MMR components is typically followed by a late positive‐going MMR, commonly associated with the well‐known P3, which occurs around 250–500ms and emerges across different senses in a modality‐independent manner (as reviewed elsewhere, e.g., Polich 2007; Friedman et al. 2001). While an enhanced P3 in response to somatosensory deviation likely involves attentional processes, the somatosensory MMN can be elicited in pre‐attentive conditions where participants attend to another primary task, as reported in many sMMN studies (Kekoni et al. 1997; Shinozaki et al. 1998; Akatsuka et al. 2005; Restuccia et al. 2007; Restuccia et al. 2009; Chen et al. 2008; Hu et al. 2013; Strömmer et al. 2014; Fardo et al. 2017; Shen et al. 2018a; Shen et al. 2018b; He et al. 2020; Xu et al. 2021; Spackman et al. 2007; Butler et al. 2012; Akatsuka, Wasaka, Nakata, et al. 2007; Akatsuka, Wasaka, Nakata, Kida, and Kakigi 2007; Spackman et al. 2010; Zhang et al. 2019; Andersen and Lundqvist 2019) which is in correspondence with auditory MMN. However, the attentional focus is also known to increase the sMMN amplitude (Hu et al. 2013; He et al. 2020) and to facilitate processing of MMR in the somatosensory system more generally (Fardo et al. 2017; He et al. 2020). Nevertheless, the occurrence of sMMN independent of attentional requirements is comparable to the auditory and visual modality and suggests a similar role of the sMMN for automatic change detection in somatosensation.

Despite sensor‐level differences, studies investigating sMMN in humans using source reconstruction of EEG or MEG signals (Naeije et al. 2016; Naeije et al. 2018; Fardo et al. 2017; Hautasaari et al. 2019; Xu et al. 2021; Spackman et al. 2010; Ostwald et al. 2012; Gijsen et al. 2021; Grundei, Schröder, et al. 2023; Andersen and Lundqvist 2019), intracranial electrocorticography (ECoG) recordings (Spackman et al. 2010; Butler et al. 2011) and functional magnetic resonance imaging (fMRI) (Chen et al. 2008) overwhelmingly suggest that it is generated cortically, primarily in somatosensory cortices. Comparing intracranial recordings and topographical distributions of EEG between an auditory and a somatosensory oddball task with duration deviants, Butler et al. (2011) showed that sMMN has generators in the postcentral gyrus (S1), whereas the auditory MMN was generated in the superior temporal gyrus (auditory cortex), suggesting that duration deviation is detected in a sensory specific manner. Similarly, in another ECoG study, Spackman et al. (2010) recorded a sMMN component from electrodes over S1 peaking around 140 ms followed by a second positive component around 200 ms likely involving S2, in line with source‐modelling studies showing S2 involvement in sMMN generation between 100 and 200 ms (Naeije et al. 2018; Andersen and Lundqvist 2019). Additionally, the authors identified a pre‐frontal component (in the middle‐frontal gyrus) around 180 ms, indicating a sensory‐frontal signal cascade underlying the sMMN which would be in line with findings from the auditory modality (e.g., Garrido, Kilner, Kiebel, and Friston 2009; El Karoui et al. 2015) and is supported by other studies on somatosensory MMR, which have indicated an involvement of pre‐frontal generators (Chen et al. 2008; Fardo et al. 2017; Spackman et al. 2010; Grundei, Schröder, et al. 2023; Allen et al. 2016). As mentioned above, in contrast to the auditory MMN, somatosensory MMR has been continuously reported prior to 100 ms. Multiple MEG (Akatsuka, Wasaka, Nakata, et al. 2007; Akatsuka, Wasaka, Nakata, Kida, and Kakigi 2007) and EEG (Gijsen et al. 2021) studies have linked early MMR components (< 100 ms) to a dipole in S1 and a later component (150–250 ms) to combined dipoles in S1 and S2, which would be in line with a processing hierarchy in the somatosensory system. Two other MEG studies using sensor and source level analyses support these findings, again showing two MMR components around ~50 ms and ~150 ms, respectively (Hautasaari et al. 2019; Xu et al. 2021). However, one study found the early component to be generated in S1 and the later component in S2, with sustained involvement of S1 only during tactile (and not electrical) stimulation (Hautasaari et al. 2019), whereas the other one found S1 and S2 activity for both components during electrical stimulation (Xu et al. 2021).

Animal studies suggest that responses resembling the somatosensory MMN may also be observed in non‐human brains, with evidence from rodents and rabbits supporting a central role for somatosensory cortex (Astikainen et al. 2001; Musall et al. 2017; English et al. 2023), comparable to human findings. This parallels work in the auditory modality, where animal studies have demonstrated MMN in both primary and secondary auditory cortices (Csépe 1995; Nieto‐Diego & Malmierca, 2016). In the somatosensory system, evidence has been reported for S1 involvement (Musall et al. 2017) as well as combined S1 and S2 activation (English et al. 2023), although many studies on rodents have concentrated on earlier stages along the thalamo‐cortical pathway up to and including S1 (e.g., Petersen 2007; Maravall et al. 2007; Lampl and Katz 2017). A key question is whether enhanced responses to deviant stimuli in these pathways depend on the probabilistic sequence context and reflect genuine deviance detection or rather reflect differences between adapted neuronal population responses to any repeated input—a phenomenon often explained by neuronal fatigue mechanisms (sensory adaptation) (Musall et al. 2017; Han et al. 2023). This debate, which is outlined in more detail in the following section, closely mirrors discussions in auditory MMN research (e.g., Harms et al. 2016; Ross and Hamm 2020; Carbajal and Malmierca 2018; Näätänen et al. 2005; May and Tiitinen 2010). Particularly at later latencies (> 100 ms), cortical responses in rodents have been linked more directly to genuine deviance detection (Musall et al. 2017; English et al. 2023; Han et al. 2023) and proposed as potential analogues of the human sMMN (Musall et al. 2017), similar to what has been argued for the auditory MMN in rats (e.g., von der Behrens et al. 2009). Yet, given the processing differences across species, the degree of correspondence of these responses remains uncertain and translational claims should therefore be made with caution. The following sections review evidence on potential mechanisms underlying the sMMN in humans and animals and highlight key gaps that call for further investigation.

## 2 Mechanisms of sMMN Generation

Different theories of MMN generation in the auditory modality have been proposed over decades of research and are continuously developed and debated. Early MMN theories assumed the component to be a comparative response signaling the divergence of sensory input from a sensory memory trace (Winkler et al. 1992; Näätänen et al. 1993), potentially serving as a saliency detector for the sensory environment registering changes in physical stimulus features (Sokolov 1963). Later, model‐based formulations of MMN generation assumed a predictive perceptual model based on abstractions of the sensory stream (Winkler 2007). Following this idea, MMN has become a focal point for investigating theories of predictive processing and perceptual inference (e.g., Friston 2005; Garrido, Kilner, Stephan, and Friston 2009; Winkler et al. 2009; Winkler and Czigler 2012; Heilbron and Chait 2018) as it is proposed to signal a discrepancy between model‐based predictions and the current sensory input, leading to model adjustments that improve future predictions.

While model‐based hypotheses highlight the relevance of top‐down predictions, adaptation‐based accounts of MMN generation have argued that observed differences between standard and deviant responses are rather explained purely bottom‐up by neuronal attenuation (or fatigue) to a repeated stimulus (e.g., May and Tiitinen 2010; May et al. 1999; Jääskelainen et al. 2004). Such sensory adaptation produces MMN‐like responses through differential adaptation across neuronal populations. Neurons repeatedly activated by the standard stimulus reduce their firing due to general fatigue, while a separate neuron population responding to a deviant stimulus remains relatively unadapted, leading to an apparent enhancement of the deviant response. Sensory adaptation is commonly observed on different levels of the neuronal circuitry (see Whitmire and Stanley (2016) for review). In contrast, *stimulus‐specific* adaptation (SSA) refers to reduced responses of single neurons (or neuronal channels) that are selective to repeated occurrences of a particular stimulus feature, while the response to other features remains comparatively preserved. Most research on sensory adaptation and SSA has been conducted in animals (primarily rodents), given the necessity of invasive methods for fine‐grained neuronal recordings, and SSA has been suggested as an underlying neuronal mechanism of auditory MMN in the animal model (Ulanovsky et al. 2003; Nelken and Ulanovsky 2007; Yu et al. 2009). Generally, SSA is known to contribute to the encoding of environmental statistics by selectively reducing neuronal responses to frequently occurring stimuli and thus effectively filtering out redundant information (Barlow 1961; Wark et al. 2007). In turn, this adaptive filtering mechanism likely allows the brain to prioritize the processing of stimuli that deviate from the expected statistical structure, facilitating a more efficient and accurate representation of the sensory environment. Therefore, across sensory domains, SSA has been suggested to be an efficient mechanism for the encoding of stimulus properties and their statistical distributions (Maravall et al. 2007; Barlow 1961; Wark et al. 2007; Khouri and Nelken 2015; Smirnakis et al. 1997; Fairhall et al. 2001; Adibi et al. 2013).

Classically, MMN has been investigated using the oddball paradigm, in which a sequence of repeated standard stimuli is occasionally interrupted by a rare deviant. However, in this paradigm, deviant stimulus changes are directly coupled to a specific stimulus property. To isolate the change response per se, studies in the auditory modality have switched the roles of standard and deviant features within a stimulus sequence (“roving‐oddball paradigm,” e.g., Cowan et al. 1993; Baldeweg et al. 2004) or in different experimental blocks (“block‐flip paradigm,” e.g., Harms et al. 2014; Fishman 2014) and averaged responses to deviants and standards independent of their feature to obtain MMN signatures. Various other control sequences have been established to investigate whether MMN reflects a response triggered by rare changes violating a regularity, indicating prediction mismatch, or rather different levels of adaptation between standards and deviants. Evidence against sensory adaptation in the auditory domain is provided, for instance, by both human (Fishman 2014; Ruhnau et al. 2012) and rodent research (Harms et al. 2016; Taaseh et al. 2011) showing increased deviant responses when presented within an oddball sequence compared to when presented alone with the same occurrence frequency (“deviant‐alone” control). Another well‐established protocol includes “many‐standard” controls (e.g., Schroger and Wolff 1996; Jacobsen and Schroger 2001) in which rare deviants presented within an oddball sequence (with, e.g., *p* = 0.2) are compared to stimuli presented within sequences of “many‐standards” in which each stimulus is equally rare (e.g., 5 different standards are presented with *p* = 0.2 each). Another paradigm which is particularly well suited to control for adaptation and stimulus related processing is the omission paradigm in which stimuli are occasionally omitted from a regular sequence or an expected stimulus pattern. Auditory omission paradigms have thus been used to elicit responses without bottom‐up input (Yabe et al. 1997; Auksztulewicz et al. 2023; Lao‐Rodriguez et al. 2023; Awwad et al. 2023; Tervaniemi et al. 1994; Hughes et al. 2001; Bendixen et al. 2009; Salisbury 2012; Chennu et al. 2016; SanMiguel, Saupe, and Schröger 2013; SanMiguel, Widmann, et al. 2013; Wacongne et al. 2011; Dercksen et al. 2020; Dercksen et al. 2022) and might be termed omission MMN if the response, time‐locked to the expected regular stimulus, occurs within the MMN time window (e.g., Yabe et al. 1997; Tervaniemi et al. 1994; Hughes et al. 2001; Salisbury 2012; Chennu et al. 2016; Prete et al. 2022). Moreover, the idea that the auditory MMN reflects a rule‐based relationship between features (Paavilainen et al. 1999; Paavilainen et al. 2007; Paavilainen 2013; Näätänen et al. 2001; Näätänen et al. 2010) has become evident in experiments in which a basic pattern is established by the sequential presentation of two alternating tone pitches and MMN is triggered by occasional repetitions (Nordby et al. 1988; Ritter et al. 1992; Alain et al. 1994; Horvath and Winkler 2004; Macdonald and Campbell 2011). Like omission responses, the elicitation of MMN by these “unexpected repetitions” cannot be easily explained by sensory adaptation to a repeated stimulus. Similarly, MMN was found in response to rare repetitions in an otherwise descending sequence of tones (Tervaniemi et al. 1994) or in a presentation of a descending tone pair in a sequence of ascending pairs (Paavilainen 2013; Saarinen et al. 1992; Ruusuvirta et al. 2007), indicating a form of abstraction from a detected rule or regularity underlying the sound presentation. It has thus been argued that “true deviance detection” is the distinguishing feature of MMN (e.g., Harms et al. 2016; Ross and Hamm 2020; Shiramatsu and Takahashi 2021), highlighting the idea that the response is not merely detecting stimulus feature changes but rather reflects surprise about an unexpected event based on the stimulus history. In rodent models, signatures of true auditory deviance detection have been reported at varying latencies between 50 and 150 ms (Harms et al. 2016), whereas sensory adaptation is particularly known to dominate prior to 50 ms.

Overall, current auditory research provides compelling evidence that true deviance detection involves the use of stimulus predictions at the macroscopic level (see, e.g., Heilbron and Chait 2018). While SSA operates at the level of individual neurons or local circuits and reflects selective adaptation to repeated stimulus features, it should be noted that SSA has also been suggested to reflect a form of local, circuit‐level prediction, whereby neurons adjust their responsiveness based on recent stimulus history (Carbajal and Malmierca 2018). However, this differs from the broader, hierarchical predictive mechanisms implicated in true deviance detection. Nevertheless, the interpretation of the auditory MMN remains debated (e.g., Garrido, Kilner, Stephan, and Friston 2009; Harms et al. 2016; Carbajal and Malmierca 2018; May and Tiitinen 2010; May 2021; Fitzgerald and Todd 2020), and extending investigations to the somatosensory modality offers the opportunity to examine sequential sensory processing in a different system with unique properties, potentially revealing whether predictive or adaptation‐based mechanisms generalize across sensory domains.

### 2.1 Sensory and Stimulus‐Specific Adaptation in the Somatosensory System

Sensory adaptation in the somatosensory system can already emerge peripherally, through adaptation in mechanoreceptor channels (SA1, SA2, RA1, RA2) or reduced responsiveness in early thalamocortical relays (Delhaye et al. 2018) (Figure 1B). In rodents, the somatosensory domain provides a particularly well‐suited system for investigating mechanisms in the underlying neuronal circuitry of stimulus processing and corresponding behaviors (Petersen 2007). Akin to the movement of human fingertips across a surface (“active touch”), rodents rely heavily on their vibrissa system to gather information (Figure 1C) (Berg and Kleinfeld 2003; Voigts et al. 2008). Triggered by mechanical movement of the whiskers, activity is projected along the trigeminal lemniscal pathway through the thalamus to the whisker representation in S1 (Figure 1C–E). Somatosensory processing in the ventral posterior medial nucleus of the thalamus (VPM) reflects the topographic organization of S1 which, in rodents, provides a precise delineation of individual whisker function in discrete anatomical units (barrels in the cortex, barreloids in the thalamus; Figure 1E). Sensory adaptation has been found along all relays of the afferent lemniscal and paralemniscal somatosensory pathways of rodents, including the trigeminal complex, VPM, POm, and whisker S1 (Lampl and Katz 2017). In fact, it has been shown that the two pathways complement each other to faithfully encode a wide range of tactile intensities by means of sensory adaptation (Mohar et al. 2015). Already at the earliest processing stages, sensory adaptation profiles in lemniscal and paralemniscal pathways were shown to differ depending on the contexts given by repeated stimulation with different intensities. While lemniscal neurons of the principal nucleus of the trigeminal nerve in the brainstem (PrV) encoded stimuli more accurately under high‐intensity conditions, paralemniscal neurons of the interpolaris subnucleus of the spinal trigeminal nucleus in the brainstem (SpVi) did so under low‐intensity conditions. Further, it has been indicated that sensory adaptation in VPM and the cortex is actively regulated by circuits in the brainstem. While little sensory adaptation was found in trigeminal neurons, it was present downstream in VPM and the cortex where it was found to be reduced for stronger intensity stimuli, contrary to traditional models of synaptic depression (Ganmor et al. 2010). Overall, it has been argued that adaptation is not simply a side effect of neural fatigue but rather an active coding strategy since it enhances the temporal and spatial context encoding of sensory inputs, particularly at the population level (Liu et al. 2017). The somatosensory stimulus sequence history can be reflected by both locally emerging adapted firing rates and by ‘inherited’ sensory adaptation from upstream stages (Mohar et al. 2015; Ganmor et al. 2010; Liu et al. 2017; Jubran et al. 2016). Therefore, subcortical (somato‐)sensory adaptation can already contribute to the encoding of complex sequence information through brainstem‐thalamic transformations and corticothalamic modulations, which primarily reflect adjustments to repeated input but potentially provide a basis for more selective, feature‐specific mechanisms such as SSA.

Like sensory adaptation, SSA has been reported at various stages of the somatosensory pathways. Already at the level of the brainstem, in PrV neurons, SSA was found for repetitive whisker stimulation (Jubran et al. 2016). However, this specificity was lost at the thalamic gate VPM, where the response became equally suppressed for any whisker stimulation, indicating that the thalamus may play a role in scaling down overall sensory input rather than encoding specific sensory features. Additionally, top‐down influences from cortical layer 6 of whisker S1 have been shown to dynamically modulate VPM activity (involving the thalamic reticular nucleus), likely refining both the precise relay of lemniscal signals and the integrative processing of paralemniscal inputs (Dimwamwa et al. 2024; Mease et al. 2014; Voigts et al. 2020). Cortical SSA has been reported for the processing of repetitive somatosensory stimuli with multiple features and a wide range of repetition rates (Musall et al. 2017; Katz et al. 2006) and in whisker S1, SSA has been shown to be reflective of stimulus feature information and their statistical distributions (Maravall et al. 2007; Diaz‐Quesada and Maravall 2008).

Building on the encoding of stimulus features and their redundancy, SSA also plays a critical role in detecting changes within the sensory environment. Current evidence suggests that SSA across different levels of the thalamocortical loop captures stimulus sequence properties, thereby shaping later stages of cortical processing and influencing learning and behavior. Evidence for behavioral relevance of SSA for somatosensory change detection was provided by Musall et al. (2017) by preventing SSA through optogenetic stimulation of whisker inputs without actual whisker stimulation. The authors found that, while behavioral performance improved for frequency discrimination, rats performed better in a change detection task when actual whisker stimulation—accompanied by SSA—was present. Strikingly, the whisker‐driven behavior was replicated only when optogenetically evoked spikes were manipulated such that they mimicked SSA. Behavioral relevance for deviance detection in a repetitive stream of standard stimuli has also been indicated in a somatosensory oddball paradigm by showing that the extent of SSA compared to the increased response to deviants in multi‐unit activity (MUA) and local field potentials (LFP) of whisker S1 was reflective of distraction by deviant stimulation from a primary visual task (Ghasemi Nejad et al. 2023). The authors showed that this behavioral effect specifically correlated with SSA in deeper cortical layers and, moreover, that layer‐specific SSA reduced with increasing experience, indicating an influence of sequence learning on these responses.

Several studies have provided evidence for layer specificity of SSA in the somatosensory system. Musall et al. (2017) reported that SSA in response to oddball sequences was strongest in supra‐ and infragranular layers of S1 barrel cortex, whereas only weak SSA was found in input layer 4, suggesting that adaptation in this case was primarily cortically generated at early stages of somatosensory processing. The relevance of cortical, layer‐specific interaction in the somatosensory system was also demonstrated by research highlighting the role of L6 and L2/3 neurons for deviant processing and predictive models (Voigts et al. 2020). It has been shown that receptive fields in L2/3 code for multi‐whisker correlation patterns (Estebanez et al. 2016) while L6 is a major cortico‐thalamic feedback relay integrating thalamic and cortical inputs (Zhang and Deschenes 1998; Thomson 2010) and neurons of L6 have relatively specific receptive fields (Velez‐Fort et al. 2014) which are not strongly driven by sensory inputs (Lee et al. 2008) but modulate sensory gain (Olsen et al. 2012).

### 2.2 Evidence for True Deviance Detection

As described above, true deviance detection can be isolated by comparing MMR in a variety of paradigms. Although sMMN has been primarily reported in response to oddball sequences, some studies have controlled for stimulus confounds by switching the roles of standards and deviants in roving‐oddball sequences (He et al. 2020; Ostwald et al. 2012; Gijsen et al. 2021; Grundei, Schröder, et al. 2023; Duerler et al. 2021) and block‐flip paradigms (Xu et al. 2021; Zhang et al. 2019). Other studies have compared deviant responses to deviant‐alone (Kekoni et al. 1997; Shinozaki et al. 1998; Restuccia et al. 2007; Restuccia et al. 2009; Astikainen et al. 2001) or many‐standard control protocols (Musall et al. 2017; Han et al. 2023) as well as omission paradigms (Naeije et al. 2018; Andersen and Lundqvist 2019; Dercksen et al. 2024) to provide evidence for true deviance detection.

Initial evidence for an omission response in the somatosensory domain has been provided by a study showing a somatosensory P100 and N140 response complex time‐locked to the rare offset of a train of repetitive stimuli (Yamashiro et al. 2008). While these findings suggest a somatosensory response to a physically absent stimulus and were found to be identical for attended and unattended stimulus trains, the response is more likely a form of offset response rather than a reflection of expectation mismatch. Since omission paradigms like these often require short inter‐stimulus intervals (ISI < 500 ms; May and Tiitinen 2010; Yabe et al. 1997), corresponding omission responses might thus rely on a form of (neuronal) entrainment mechanism. Interestingly, however, the same somatosensory EEG components were identified in an omission paradigm using self‐generated tactile stimuli with ISI between 600 and 1200 ms (Dercksen et al. 2024). This study showed that, if an action (button press) was reliably coupled with a tactile stimulus (in 88% of trials) and thus predictable, somatosensory omission responses were observed following occasional omissions of the tactile stimulus. In contrast, no omission response occurred if the coupling was not predictable (i.e., occurred in 50% of trials). The same group also reported an omission response in pupil dilation to somatosensory stimulus omissions in the same paradigm with ISI of 3000 ms (Dercksen et al. 2023). These results suggest a sensitivity of omission responses to stimulus predictability, similar to findings in the auditory modality (see, e.g., SanMiguel, Saupe, and Schröger 2013; Dercksen et al. 2020). Evidence for an omission sMMN proper was provided by Naeije et al. (2018) (ISI = 500 ms) and Andersen and Lundqvist (2019) (ISI = 3000 ms) who combined MEG measurements with source modelling. Both studies showed an omission sMMN between 100 and 200 ms generated in S2. Moreover, Andersen and Lundqvist (2019) showed differential oscillatory modulations for the omission sMMN and stimulus repetitions. While the omission response, thought to reflect prediction mismatch, was accompanied by power increases in the gamma band localized to the insular cortex, stimulus repetitions (i.e., redundancy) exhibited increased beta‐band frequency localized to the parietal cortex. These observations are in line with research suggesting an asymmetric relationship of sensory predictions and their violation carried by beta and gamma band oscillations, respectively (Bastos et al. 2015) (for reviews, see, e.g., Arnal and Giraud 2012; Friston 2019).

**FIGURE 3:**
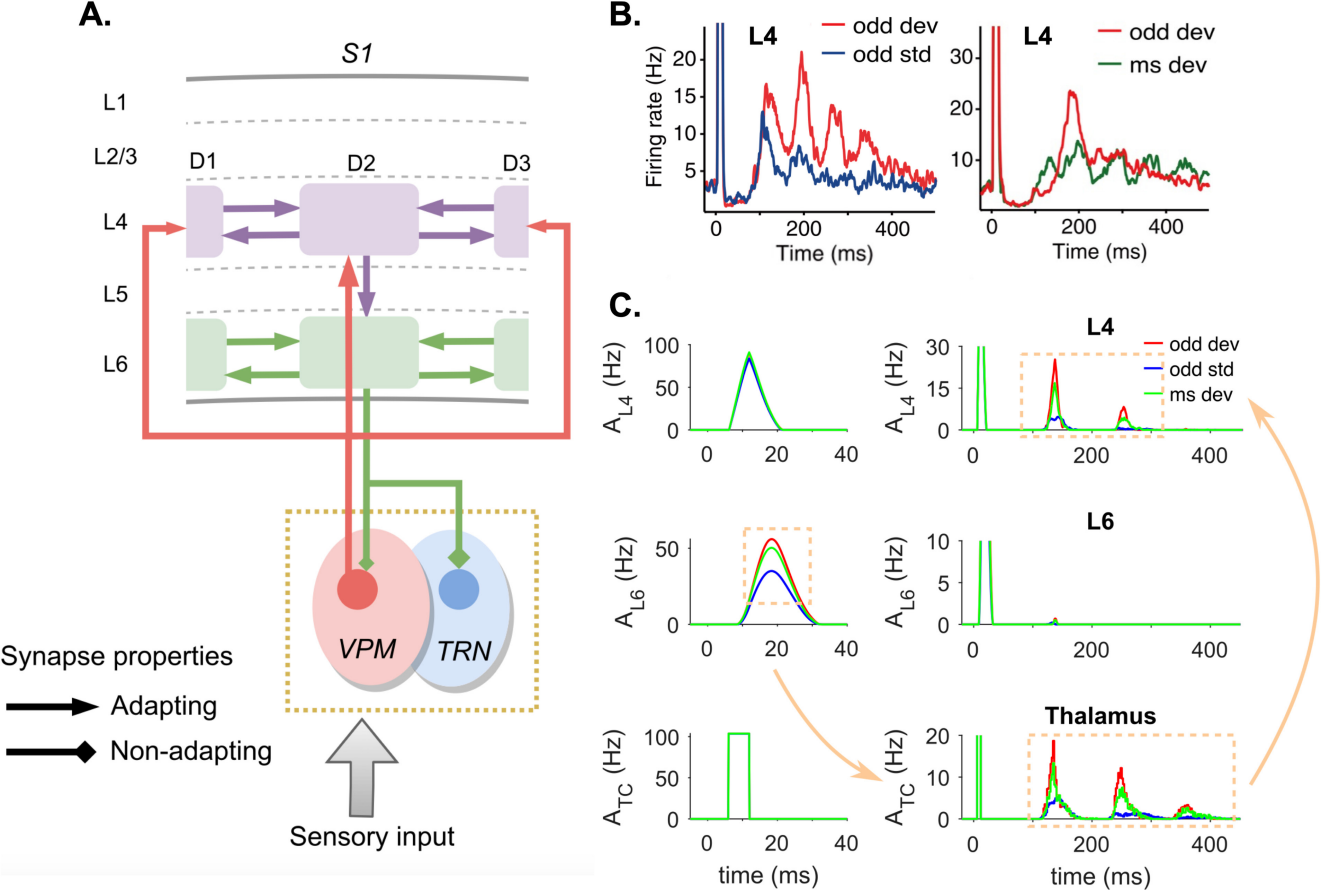
A minimal thalamocortical loop model. (A) The S1 barrel cortex with barreloids for whiskers D1–D3. Layer 4 (L4) receives thalamic inputs (strongest to the principal whisker barreloid, indicated by arrow size) and projects to L6 which has modulating feedback projections to the ventral posterior medial (VPM) and thalamic reticular nuclei (TRN) with strength of modulation indicated by diamond size). (B) Empirical recordings from multi‐unit activity (MUA) of L4 show increased late true deviance detection in response to oddball deviants (red) compared to standards from oddball (blue) and many‐standards paradigms (green). *Original data from* Musall et al. (2017). (C) Modelled population activity shows initiation of SSA and true deviance detection in early responses of L6 which are subsequently enhanced by thalamic oscillations and ultimately induce late true deviance detection in cortex via feedback loop. *Figure adapted from* Han et al. (2023) *with permission (CC BY 4.0)*.

In the animal model, Astikainen et al. (2001) showed that deviant responses recorded in the somatosensory cortex (and somatosensory projection areas of the cerebellum) of rabbits were larger for deviants compared to standards and in light of the deviant‐alone condition both at early (20–40 ms) as well as later (100–120 ms) latencies. On the other hand, using the many‐standards control, Musall et al. (2017) reported a late signal starting from around 100 ms in a subset of neurons in the granular layer of rats which showed true deviance detection properties (Figure 3B), while earlier responses found in supra‐ and infragranular layers of S1 corresponded to SSA. In contrast to earlier SSA‐related effects, the true deviant response was expressed among other measures as a mid‐latency negativity in the LFP between 150 and 250 ms. While these responses show an apparent resemblance to the human sMMN, it remains uncertain whether the processing latencies in the somatosensory system are truly comparable and whether they can be considered a rodent analogue.

### 2.3 True Deviance Detection in the Thalamocortical Loop

Evidence from both auditory as well as somatosensory MMN research indicates that true deviance detection and SSA are both critical functionally intertwined processes for shaping the encoding of stimulus information along the thalamocortical pathway. Recently, Han et al. (2023) have thus proposed a computational model of the thalamocortical loop of the somatosensory system to account for early signals of SSA as well as the later occurrence of true deviance detection (Musall et al. 2017; English et al. 2023). The authors developed a minimalistic model (Figure 3) consisting of a hierarchical network including the barrel cortex (S1) and thalamic relays (VPM and reticular nucleus). In similarity to other models of SSA, the model is based on short‐term synaptic depression. However, for the emergence of deviance detection properties, it critically depends on network‐level interactions. According to the model, neurons in cortical layer 6 adapt to repeated stimulation, resulting in reduced population activity at short latencies (~20 ms). As the activity to a rarely presented deviant stimulus is comparably higher, this difference is projected to the thalamus where it is amplified by oscillatory interactions between the thalamic nuclei. Subsequent thalamocortical projections forward the oscillatory activity to cortical layer 4 where it can be observed as a late response (~120 ms). Using (among others) the many‐standards control protocol, the authors present simulated model responses to oddball sequences and show that this mid‐latency signal reflected true deviance detection and context sensitivity. As such, this minimal model offers valuable insights into how the thalamocortical pathway may contribute to both early SSA and later‐stage deviance detection, bridging a functional link between both in the somatosensory system.

### 2.4 Probabilistic Sequence Learning and Surprise

**FIGURE 4:**
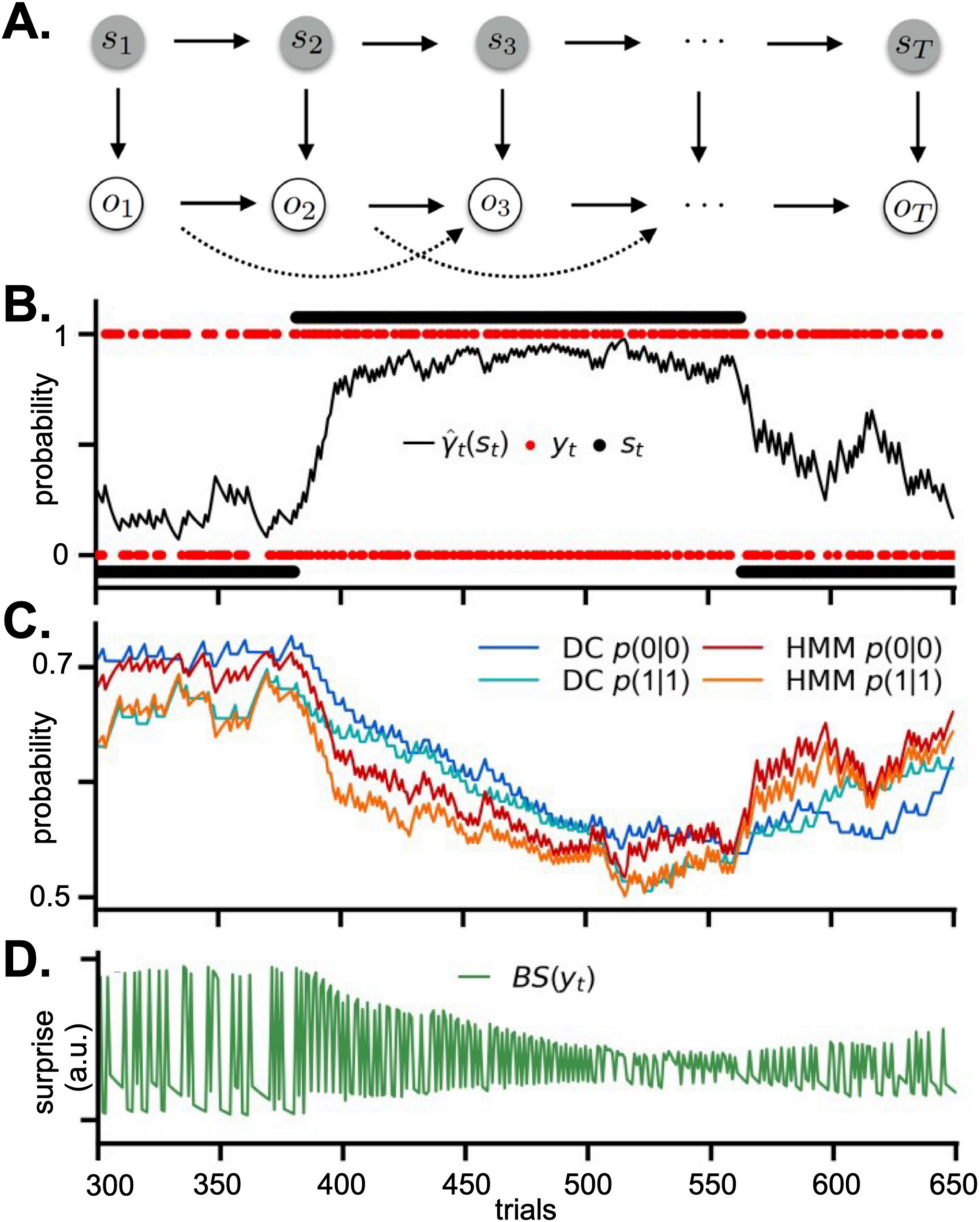
Probabilistic sequence learning. (A) Stimulus sequences can be described as Markov chains. Example schema of hidden states (s) and observations (o) evolving probabilistically from t=1 to t=T according to specified transition probabilities. Thick horizontal arrows reflect state and observation transitions, respectively. Dashed arrows reflect influence by second order transition probability. Thick downward arrows reflect emission probability of observations by hidden states. (B) Example sequence of observations (red dots) and hidden states (black dots; $s_{0}$ produces slow switching observations with change probability p=0.3; $s_{1}$ produces fast switching observations with change probability p=0.5). The black line corresponds to the hidden state inference of a hidden Markov model (HMM; plotted is the probability of $s_{1}$). (C) Model trajectories of estimated observation repetition probabilities of a non‐hierarchical Bayesian model with forgetting (Dirichlet‐Categorical model; DC) and an HMM with explicit modelling of hidden state dynamics. (D) Corresponding surprise read‐out of model probabilities of the DC model, exemplified by Bayesian surprise (BS). *Figure adapted from* Gijsen et al. (2021) *with permission (CC BY 4.0)*.

Both true deviance detection and SSA, likely contributing on different levels to the auditory MMN, have been hypothesized to reflect processes related to predictive coding and probabilistic inference more broadly (for reviews, see, e.g., Friston 2005; Carbajal and Malmierca 2018; Auksztulewicz and Friston 2016). As recently summarized by Schröger et al. (2023), various experimental stimulus sequences employed in MMN research can be described as Markov chains governed by transition probabilities, i.e., the probability of transitioning from one sequence item to the next. Interestingly, research in the auditory modality has identified transition probability as one of the key properties for processing sequential sensory input and suggested it to be an important building block for more complex processes, including perception and language (see Dehaene et al. 2015 for a review). Current evidence from computational modelling supports the view that sequential sensory inference, hypothesized to underlie the auditory MMN, is based on transition probability learning (Meyniel et al. 2016; Maheu et al. 2019). Under the assumption that MMN is reflective of an underlying inference process on the statistical structure of the environment (Friston 2005; Garrido, Kilner, Stephan, and Friston 2009; Garrido et al. 2013), computational models performing Bayesian inference can model the trial‐to‐trial variation of responses to probabilistic input sequences (Figure 4). Research on MMN thus increasingly shifted the focus away from average‐based analyses towards such single‐trial modelling of mismatch dynamics in the auditory (e.g., Maheu et al. 2019; Lieder et al. 2013; Weber et al. 2020; Lecaignard et al. 2021, 2022; Poublan‐Couzardot 2023; Modirshanechi et al. 2019), the visual (e.g., Stefanics et al. 2018; Schlossmacher et al. 2022) as well as the somatosensory modality (Ostwald et al. 2012; Gijsen et al. 2021; Grundei, Schröder, et al. 2023). These models iteratively quantify *prediction mismatch* about new incoming observations by different applications of prediction error and information theoretic measures of surprise. In this context, surprise corresponds to a computational quantity which reflects expectation mismatch in probabilistic terms (see Modirshanechi et al. 2022 for a review of different surprise quantifications).

In the somatosensory modality, Ostwald et al. (2012) provided first evidence for a reflection of surprise in single‐trial dynamics of the sMMN. The authors showed that their EEG data were best explained by a Bayesian observer model with limited sequence integration (i.e., forgetting of past observations), compared to simple change‐detection or linear adaptation models. Their results indicated that S2 encoded surprise at around 140 ms. Employing a larger model space, Gijsen et al. (2021) extended these findings by comparing different surprise quantifications as well as inference strategies to model sequential observations. Like the previous study, the authors employed a roving‐oddball paradigm in which stimuli alternate between two features with a higher probability to repeat (standard) than change (deviant), but with equal probability of each feature across the sequence (*p* = 0.5). Their model comparison results showed that a model differentiating unique stimulus transitions best explained sMMN compared to a model tracking stimulus alternation (i.e., changes) or stimulus identity. Moreover, to be able to account for changing environmental statistics in the sequences (i.e., volatility), the study indicated that somatosensory inference might rely on a limited or “leaky” integration of the recent past (Ostwald et al. 2012; Gijsen et al. 2021), rather than explicit representation of hidden states. This has been argued to be biologically plausible for adaptive inference (Ryali et al. 2018; Yu and Dayan 2005; Farashahi et al. 2017; Glaze et al. 2015). Additionally, the authors provided evidence for differences between earlier and later mismatch signatures, reflecting confidence weighted surprise (found around 70–170 ms in S2) and model updating (found around 150 ms in S1 and around the P3 response), respectively. Although not assessed by means of the sMMN response, later research on somatosensory statistical learning additionally supported a central role for confidence in probabilistic inference in the somatosensory nociceptive system (Mulders et al. 2023), comparable to prior suggestions from the auditory modality (Meyniel and Dehaene 2017; Meyniel 2020). The study showed that higher confidence of participants in their predictions of stimulus transitions was reflected in stronger suppression of the somatosensory vertex potential (Mulders et al. 2023). Further support for a temporal dissection of surprise computation reflected in MMRs was given by a recent multi‐sensory study which indicated similar temporal evolution of signals from early confidence weighted surprise to later belief updating (Grundei, Schröder, et al. 2023). Using a tri‐modal version of the roving paradigm, Grundei, Schröder, et al. (2023) showed similarities of the auditory, somatosensory, and visual MMN and provided evidence that central responses (from ~150 to ~350 ms), likely originating from fronto‐parietal regions (see Grundei, Schmidt, and Blankenburg 2023), were sensitive to the probability of stimulus transitions conditional on the multi‐sensory configuration of stimuli. These results indicated a multi‐sensory inference process at mid‐latency to late processing stages, whereas earlier responses (Grundei, Schröder, et al. 2023) and responses in sensory regions (Grundei, Schmidt, and Blankenburg 2023) were more sensitive to uni‐sensory transitions indicative of a multi‐modal hierarchy of probabilistic sequence learning.

To specifically investigate responses to sensory regularities at different hierarchical levels, the local–global paradigm has been an influential experimental protocol in the auditory modality (Bekinschtein et al. 2009). In this paradigm, sequences consist of chunks of a few identical, repeating stimuli followed by one differing stimulus. Thus, the last stimulus is a local deviant while it conforms to a globally expected pattern (standard). This global regularity can be violated by occasionally presenting chunks in which the last stimulus is a local standard (instead of a local deviant). It has been shown that the repetition of a standard sound at the position where the deviant was (globally) expected elicited a P3 response, whereas no MMN was observed (El Karoui et al. 2015; Chennu et al. 2016; Wacongne et al. 2011; Bekinschtein et al. 2009; Chennu et al. 2013; Chao et al. 2018; Uhrig et al. 2014). Most likely, this P3 response to global deviants is modality independent as it was recently shown for auditory, visual, and somatosensory sequences alike (Niedernhuber et al. 2022). Similarly, in the somatosensory modality alone, the local–global paradigm has also been shown to elicit this expected pattern (Naeije et al. 2016): Local deviants elicited a sMMN‐equivalent in the MEG between 55 and 130 ms, which was localized to contralateral S2, whereas global deviants (local repetitions) showed an increased P3 response, generated by a fronto‐parietal network, with an absent sMMN. The finding of a P3 response to global regularity violations (local stimulus repetitions) indicates that the response reflects expectations on a more global scale of sequence perception. This suggests a hierarchy of MMR processing with increasing levels of information integration in line with earlier interpretations of MMN and P3 reflecting two stages of novelty processing (Friedman et al. 2001; Escera et al. 2000; Wetzel and Schröger 2014).

In line with research in humans, modulations of single‐trial responses in rat somatosensory cortex have been shown to be captured by a computational model estimating surprise about stimulus transitions (English et al. 2023). Surprise was found to correlate strongest with deviance‐related activity in both S1 and S2. Interestingly, deviant responses were particularly sensitive to sequence context, which was modulated by differences in first‐order Markov transitions. Using a variation of the many‐standards paradigm, the authors presented stimulation sequences in which three standard whiskers were each stimulated with equal probability (*p* ~ 0.32) and occasionally interrupted by a rare stimulation of a fourth deviant whisker (*p* ~ 0.03). Two types of sequences were defined by differences in the underlying transition probabilities between stimuli. In a regular sequence, the standards followed a fixed transitional structure (e.g., A‐B‐C‐A‐B‐C‐ …) and in a randomized sequence, the transitions between standards were random (e.g., A‐C‐B‐C‐A‐B‐B‐C‐A‐ …). Recordings were performed throughout the cortical layers of S1 and S2 as well as the VPM and POm of the somatosensory thalamus. In contrast to the minute differences in the thalamic nuclei and the thalamic projection layer 4, pronounced surprise responses and clear differences between both stimulation protocols were observed in the principal columns of the deviant whisker in S1 and S2. These differences were the highest in layer 6, although with opposing sign. This result indicates that the context sensitivity of true deviant responses emerges cortically in the somatosensory system. Moreover, deviant responses were found as increases in gamma power in S1 L2/3 and in alpha power in S2 L2/3 and L6, whereas responses to standards within the regular sequence elicited higher alpha/beta power in S2 L2/3 and L6. In line with previously proposed functional asymmetry of oscillatory responses in cortical regions (Bastos et al. 2015), these results indicate that S1 rather projects prediction errors via gamma band oscillations via feedforward layers 2/3, while S2 additionally encodes predictions mediated by low‐frequency oscillations in feedback layer 6. Interestingly, these responses were shown to be NMDAR‐dependent, which is also a well‐known property of the auditory MMN, in both humans and animals (Harms et al. 2016; Michie et al. 2016). English et al. (2023) showed that the somatosensory deviance‐induced negativity in LFP between 100 and 200 ms in both S1 (L5/6) and S2 (across all layers) was reduced by the NMDAR antagonist ketamine, similarly to the auditory modality. This provides additional support for a delineation of such surprising deviant responses from SSA, which is not affected by modulation of NMDAR function (Farley et al. 2010). However, future research is required to disentangle the exact mechanisms underlying NMDAR contribution, including the identification of specific receptor subtypes and interacting neurotransmitter systems. Similarly, the functional details of the reported layer‐specificity remain to be clarified. Nevertheless, these results provide further evidence that true deviance detection in the somatosensory system—reflected as surprise computation through S1–S2 interactions around 100–200 ms and involving NMDA receptor function—shows context sensitivity induced by differences in stimulus transitions. Overall, this study complements recent research in suggesting that the somatosensory system—and possibly the entire sensory system (e.g., Grundei, Schröder, et al. 2023; Dehaene et al. 2015)—is particularly sensitive to the transition probability of sequential input.

Taken together, computational approaches indicate that sMMN reflects the brain's ability to detect unexpected changes in somatosensory input, most likely functioning as a surprise signal. Some studies indicate that the processing principles share similarities with those of the auditory MMN, potentially arising from comparable underlying mechanisms that include both SSA and true deviance detection. While SSA explains how neurons reduce responsiveness to frequent stimuli to prioritize novel inputs and encode stimulus features, deviance detection signals discrepancies between predicted and actual sensory events. These mechanisms rely on a dynamic interplay between cortical and subcortical structures, and ongoing computational and neurophysiological research continues to refine our understanding of this hierarchical system.

## 3 Relevance of sMMN for Clinical and Special Populations

The pre‐attentive character of sMMN makes the response a useful tool for clinical investigations of sensory function in unresponsive or non‐verbal individuals (Näätänen 2009). Notably, it shares similarities with the auditory MMN but offers additional advantages due to its modality‐specific properties and its applicability even in the absence of intact auditory function. The mature functioning of sMMN can be already observed in healthy children (Restuccia et al. 2009; Spackman et al. 2010) and infants (Shen et al. 2020), and its alteration can provide valuable insights into sensory processing across the lifespan. For instance, sMMN to change in location is found to be decreased in amplitude in older adults (Hu et al. 2013; Strömmer et al. 2014; Strömmer et al. 2017). Although a recent MEG study did not show aging effects in sMMN using a similar paradigm, modulations were found in early somatosensory responses associated with sensory gating and a later attention shift mechanism (Pesonen et al. 2023). In an EEG study, comparisons of auditory MMN and a (positive polarity) somatosensory MMR in older adults revealed that only the somatosensory MMR showed age‐related changes (Strömmer et al. 2017), suggesting that it may be more sensitive to aging‐related brain activity alterations. Interestingly, a larger amplitude was correlated with better executive function and increased walking distance, highlighting its potential relevance to functional health in aging populations (Strömmer et al. 2017). Additionally, across the lifespan, sMMN appears to relate to physical activity levels. For example, active and inactive twins exhibited differences in dipolar source strengths in areas like the postcentral, medial frontal, and superior temporal gyri, with inactive twins showing increased dipole strengths—potentially indicating less effective somatosensory information gating (Hautasaari et al. 2017). These findings encourage the search for opportunities to use sMMN as a biomarker for sensory and functional brain changes related to aging and lifestyle.

As already suggested by Näätänen (2009), alterations of the somatosensory MMN in particular might provide comparative measures for developmental disorders and potential additional insights into neurodegenerative diseases and psychiatric conditions as these are all known to be accompanied by modulations of the auditory MMN. Given the clinical relevance of the NMDAR dependency of the auditory MMN, particularly with regard to schizophrenia and psychosis (for reviews see, e.g., Sterzer et al. 2018; Todd et al. 2023; Gutlin et al. 2024), it is interesting that a similar dependency has been reported for sMMN in mice (as described above; English et al. 2023). However, given the lack of research, it remains an open question if modulations of sMMN can be observed in schizophrenia patients and if these might be related to an altered predictive mechanism, comparable to the auditory modality. Generally, research on sMMN in a clinical context is rare. Recent studies have begun to address this gap in adults on the autism spectrum. Using a duration‐based vibrotactile oddball paradigm with neurotypical control groups, they demonstrated that autistic adults exhibit robust sMMN responses, indicating preserved temporal sensitivity to vibrotactile deviations (Isenstein, Freedman, Xu, et al. 2024; Isenstein, Freedman, Molholm, and Foxe 2024). Interestingly, these studies further showed that while passive somatosensory processing was similar to neurotypical adults, self‐generated touch elicited altered neural responses in autistic participants, highlighting a modality‐specific difference in predictive mechanisms (Isenstein et al. 2025). A recent study investigated whether auditory or somatosensory event‐related potentials (ERPs) could separate depressed and non‐depressed participant groups (Kangas et al. 2024). Neither somatosensory nor auditory MMN showed group differences, although auditory N1 in response to intensity changes separated depressive participants. However, modulations of the sMMN might be related to changes in executive function as, for instance, patients with cerebellar damage showed abnormal sMMN (Restuccia et al. 2007) and patients with dystonia showed an altered sMMN while the auditory MMN was not affected (Chen et al. 2018). Moreover, it is possible that sMMN is altered in clinical conditions related, more specifically, to changes in somatosensation such as in patients with chronic pain or with altered body representations observed in eating disorders.

In an interesting line of research, Shen and colleagues (Shen et al. 2018a, 2018b, 2020) have highlighted the potential of sMMN to provide insights into body representation and somatosensory processing. One study (Shen et al. 2018b) demonstrated the use of sMMN to study body part segmentation by showing a smaller sMMN for location deviants when the stimuli were placed on the forearm within the wrist boundary, compared to an sMMN for equidistant stimulation points separated by the wrist boundary (which is thought to be a body‐part segmentation boundary; see, e.g., de Vignemont et al. 2009). The authors argued that the increased sensory mismatch for deviant stimulation across the wrist boundary suggests a different representation of the hand as a separate body part. Using the same study protocol, another study (Shen et al. 2020) indicated that infants already show such a structured body representation at a few months of age. Moreover, a third study (Shen et al. 2018a) provided evidence for an influence of the somatotopic organization of the cortex on sMMN. The authors showed the sMMN to location deviants of different distances in somatotopic space compared to distances in physical space on the body. In a classic oddball protocol, stimulation on the lip functioned as the standard stimulus and sMMN to deviant stimulation on the neck (close bodily distance and far somatotopic distance) was compared to the sMMN to deviants on the hand (far bodily distance, close somatotopic distance). Despite being closer in physical space, the neck deviant elicited a larger sMMN than the hand deviant, suggesting that early somatosensory MMR processing is influenced by the somatotopic representation of body parts in the brain (Figure 1A). These studies highlight the need for careful design of sMMN paradigms that take the dependence of somatosensory response differences between standards and deviants on somatotopic properties into account.

Overall, sMMN offers a valuable tool for exploring sensory processing in special populations in clinical and developmental contexts. Its occurrence even under no attentional demands makes it particularly useful for assessing non‐verbal or unconscious individuals. Studies in older adults show age‐related declines in sMMN amplitude, correlating with cognitive and physical health markers, making it a potential biomarker for aging. Research has also highlighted its relevance in clinical conditions related to sensory–motor functioning and for the understanding of sensory organization in infants. Expanding studies into neuropsychiatric and pain disorders could further establish its role in diagnosing and understanding these conditions.

## 4 Conclusion and Future Directions

The study of the somatosensory MMN offers valuable insights into sensory processing, perceptual inference, and potential clinical applications. As an analogous yet distinct response to the auditory MMN, it is reliably elicited by changes in location, frequency, duration, intensity, and omissions, and is not dependent on attentional engagement. While these eliciting factors parallel those in the auditory domain, the sMMN's modality‐specific expression—shaped by somatotopic cortical maps, receptor‐specific afferent channels, and the integration of cutaneous and proprioceptive signals—reflects the unique computational demands of the somatosensory system in constructing coherent representations of touch, texture, and body position. This makes the sMMN a promising tool for probing the integrity of somatosensory pathways and cortical hierarchies, as well as for testing predictive coding mechanisms in tactile perception. At the same time, its translational potential remains to be firmly established. Future progress will require adapting well‐developed auditory MMN control paradigms to the somatosensory modality, particularly in humans. On the one hand, studies are needed to probe violations of more abstract tactile regularities—such as patterns, sequences, or object‐based predictions. At the same time, more systematic investigations of how somatosensory‐specific properties on the neuroanatomical and neurophysiological level influence the sMMN will be essential for clarifying its functional significance and potential diagnostic value.

Nevertheless, significant progress has been made in understanding the cortical and subcortical mechanisms underpinning the sMMN, with findings indicating a hierarchical processing system involving both adaptation and surprise. Computational approaches suggest that processes of probabilistic learning drive sMMN responses, and rodent models have further enhanced our understanding of the involved thalamocortical pathways. However, compared to the auditory modality, significantly less research exists on sMMN and probabilistic somatosensory stimulus processing more generally. Therefore, further studies in the somatosensory domain employing computational modelling of single‐trial responses to probabilistic input are essential to complete the picture and fully elucidate its mechanisms and implications.

Finally, we emphasize the importance of studying the specific neuronal circuits underlying sMMN in animal models. While numerous recent studies have examined the neurons and circuits involved in prediction error computation and MMN generation in auditory (e.g., Chen et al. 2015; Shymkiv et al. 2025), visual (e.g., Hamm and Yuste 2016), and sensorimotor systems (e.g., Keller et al. 2012; Jordan and Keller 2023), such data remain sparse for the somatosensory system. Future investigations into these circuits would be crucial for uncovering the generalizable principles of predictive processing (see Keller and Mrsic‐Flogel 2018).

In summary, the sMMN represents a critical window into understanding the brain's ability to detect and adapt to changes in sensory environments. Continued interdisciplinary efforts are essential to unravel its complexities and harness its potential for clinical and theoretical advancements in neuroscience. As Risto Näätänen already pointed out in 2009 (Näätänen 2009), further investigation of the sMMN and its relationship to auditory and visual MMN is needed to uncover shared computational principles across sensory systems while also clarifying modality‐specific mechanisms.

## Author Contributions

Conceptualization: MG, WB, FB. Visualization: MG. Writing (original draft): MG. Writing (review and editing): MG, WB, PA, NW, FB.

## Conflicts of Interest

The authors declare no conflicts of interest.

## Data Availability

No new data were generated or analyzed in this study. Data sharing is not applicable to this article.

## References

[ejn70301-bib-0001] Adibi, M. , J. S. McDonald , C. W. G. Clifford , and E. Arabzadeh . 2013. “Adaptation Improves Neural Coding Efficiency Despite Increasing Correlations in Variability.” Journal of Neuroscience 33, no. 5: 2108–2120. 23365247 10.1523/JNEUROSCI.3449-12.2013 PMC6619115

[ejn70301-bib-0004] Akatsuka, K. , T. Wasaka , H. Nakata , et al. 2007. “Objective Examination for two‐Point Stimulation Using a Somatosensory Oddball Paradigm: An MEG Study.” Clinical Neurophysiology 118, no. 2: 403–411. 17095288 10.1016/j.clinph.2006.09.030

[ejn70301-bib-0002] Akatsuka, K. , T. Wasaka , H. Nakata , K. Inui , M. Hoshiyama , and R. Kakigi . 2005. “Mismatch Responses Related to Temporal Discrimination of Somatosensory Stimulation.” Clinical Neurophysiology 116, no. 8: 1930–1937. 15982927 10.1016/j.clinph.2005.04.021

[ejn70301-bib-0003] Akatsuka, K. , T. Wasaka , H. Nakata , T. Kida , and R. Kakigi . 2007. “The Effect of Stimulus Probability on the Somatosensory Mismatch Field.” Experimental Brain Research 181, no. 4: 607–614. 17516059 10.1007/s00221-007-0958-4

[ejn70301-bib-0005] Alain, C. , D. L. Woods , and K. H. Ogawa . 1994. “Brain Indices of Automatic Pattern Processing.” Neuroreport 6, no. 1: 140–144. 7703403 10.1097/00001756-199412300-00036

[ejn70301-bib-0006] Allen, M. , F. Fardo , M. J. Dietz , et al. 2016. “Anterior Insula Coordinates Hierarchical Processing of Tactile Mismatch Responses.” NeuroImage 127: 34–43. 26584870 10.1016/j.neuroimage.2015.11.030 PMC4758822

[ejn70301-bib-0007] Andersen, L. M. , and D. Lundqvist . 2019. “Somatosensory Responses to Nothing: An MEG Study of Expectations During Omission of Tactile Stimulations.” NeuroImage 184: 78–89. 30213774 10.1016/j.neuroimage.2018.09.014

[ejn70301-bib-0008] Arnal, L. H. , and A. L. Giraud . 2012. “Cortical Oscillations and Sensory Predictions.” Trends in Cognitive Sciences 16, no. 7: 390–398. 22682813 10.1016/j.tics.2012.05.003

[ejn70301-bib-0009] Astikainen, P. , T. Ruusuvirta , and T. Korhonen . 2001. “Somatosensory Event‐Related Potentials in the Rabbit Cerebral and Cerebellar Cortices: A Correspondence With Mismatch Responses in Humans.” Neuroscience Letters 298, no. 3: 222–224. 11165446 10.1016/s0304-3940(00)01747-x

[ejn70301-bib-0010] Auksztulewicz, R. , and K. Friston . 2016. “Repetition Suppression and Its Contextual Determinants in Predictive Coding.” Cortex 80: 125–140. 26861557 10.1016/j.cortex.2015.11.024 PMC5405056

[ejn70301-bib-0011] Auksztulewicz, R. , V. G. Rajendran , F. Peng , J. W. H. Schnupp , and N. S. Harper . 2023. “Omission Responses in Local Field Potentials in Rat Auditory Cortex.” BMC Biology 21, no. 1: 130. 37254137 10.1186/s12915-023-01592-4 PMC10230691

[ejn70301-bib-0012] Awwad, B. , M. M. Jankowski , A. Polterovich , S. Bashari , and I. Nelken . 2023. “Extensive Representation of Sensory Deviance in the Responses to Auditory Gaps in Unanesthetized Rats.” Current Biology 33: 3024–3030.e3. 37385255 10.1016/j.cub.2023.06.013

[ejn70301-bib-0013] Baldeweg, T. , A. Klugman , J. Gruzelier , and S. R. Hirsch . 2004. “Mismatch Negativity Potentials and Cognitive Impairment in Schizophrenia.” Schizophrenia Research 69, no. 2–3: 203–217. 15469194 10.1016/j.schres.2003.09.009

[ejn70301-bib-0014] Barlow, H. B. 1961. “Possible Principles Underlying the Transformation of Sensory Messages.” Sensory Communication 1, no. 1: 217–233.

[ejn70301-bib-0015] Bastos, A. M. , V. Litvak , R. Moran , C. A. Bosman , P. Fries , and K. J. Friston . 2015. “A DCM Study of Spectral Asymmetries in Feedforward and Feedback Connections Between Visual Areas V1 and V4 in the Monkey.” NeuroImage 108: 460–475. 25585017 10.1016/j.neuroimage.2014.12.081 PMC4334664

[ejn70301-bib-0016] Bekinschtein, T. A. , S. Dehaene , B. Rohaut , F. Tadel , L. Cohen , and L. Naccache . 2009. “Neural Signature of the Conscious Processing of Auditory Regularities.” Proceedings of the National Academy of Sciences of the United States of America 106, no. 5: 1672–1677. 19164526 10.1073/pnas.0809667106 PMC2635770

[ejn70301-bib-0017] Bendixen, A. , E. Schröger , and I. Winkler . 2009. “I Heard That Coming: Event‐Related Potential Evidence for Stimulus‐Driven Prediction in the Auditory System.” Journal of Neuroscience 29, no. 26: 8447–8451. 19571135 10.1523/JNEUROSCI.1493-09.2009 PMC6665649

[ejn70301-bib-0018] Berg, R. W. , and D. Kleinfeld . 2003. “Rhythmic Whisking by Rat: Retraction as Well as Protraction of the Vibrissae Is Under Active Muscular Control.” Journal of Neurophysiology 89, no. 1: 104–117. 12522163 10.1152/jn.00600.2002

[ejn70301-bib-0019] Butler, J. S. , J. J. Foxe , I. C. Fiebelkorn , M. R. Mercier , and S. Molholm . 2012. “Multisensory Representation of Frequency Across Audition and Touch: High Density Electrical Mapping Reveals Early Sensory‐Perceptual Coupling.” Journal of Neuroscience 32, no. 44: 15338–15344. 23115172 10.1523/JNEUROSCI.1796-12.2012 PMC3664421

[ejn70301-bib-0020] Butler, J. S. , S. Molholm , I. C. Fiebelkorn , M. R. Mercier , T. H. Schwartz , and J. J. Foxe . 2011. “Common or Redundant Neural Circuits for Duration Processing Across Audition and Touch.” Journal of Neuroscience 31, no. 9: 3400–3406. 21368051 10.1523/JNEUROSCI.3296-10.2011 PMC3204366

[ejn70301-bib-0021] Carbajal, G. V. , and M. S. Malmierca . 2018. “The Neuronal Basis of Predictive Coding Along the Auditory Pathway: From the Subcortical Roots to Cortical Deviance Detection.” Trends in Hearing 22: 2331216518784822. 30022729 10.1177/2331216518784822 PMC6053868

[ejn70301-bib-0022] Carvell, G. E. , and D. J. Simons . 1990. “Biometric Analyses of Vibrissal Tactile Discrimination in the rat.” Journal of Neuroscience 10, no. 8: 2638–2648. 2388081 10.1523/JNEUROSCI.10-08-02638.1990 PMC6570272

[ejn70301-bib-0023] Chao, Z. C. , K. Takaura , L. Wang , N. Fujii , and S. Dehaene . 2018. “Large‐Scale Cortical Networks for Hierarchical Prediction and Prediction Error in the Primate Brain.” Neuron 100, no. 5: 1252–1266 e3. 30482692 10.1016/j.neuron.2018.10.004

[ejn70301-bib-0024] Chen, I. W. , F. Helmchen , and H. Lutcke . 2015. “Specific Early and Late Oddball‐Evoked Responses in Excitatory and Inhibitory Neurons of Mouse Auditory Cortex.” Journal of Neuroscience 35, no. 36: 12560–12573. 26354921 10.1523/JNEUROSCI.2240-15.2015 PMC6605391

[ejn70301-bib-0025] Chen, J. C. , D. Hämmerer , and K. D'Ostilio . 2014. “Bi‐Directional Modulation of Somatosensory Mismatch Negativity With Transcranial Direct Current Stimulation: an Event Related Potential Study.” Journal of Physiology 592, no. 4: 745–757. 24366257 10.1113/jphysiol.2013.260331 PMC3934712

[ejn70301-bib-0026] Chen, J. C. , A. Macerollo , A. Sadnicka , et al. 2018. “Cervical Dystonia: Normal Auditory Mismatch Negativity and Abnormal Somatosensory Mismatch Negativity.” Clinical Neurophysiology 129, no. 9: 1947–1954. 30015084 10.1016/j.clinph.2018.05.028

[ejn70301-bib-0027] Chen, T. L. , C. Babiloni , A. Ferretti , et al. 2008. “Human Secondary Somatosensory Cortex Is Involved in the Processing of Somatosensory Rare Stimuli: an fMRI Study.” NeuroImage 40, no. 4: 1765–1771. 18329293 10.1016/j.neuroimage.2008.01.020

[ejn70301-bib-0029] Chennu, S. , V. Noreika , D. Gueorguiev , et al. 2013. “Expectation and Attention in Hierarchical Auditory Prediction.” Journal of Neuroscience 33, no. 27: 11194–11205. 23825422 10.1523/JNEUROSCI.0114-13.2013 PMC3718380

[ejn70301-bib-0028] Chennu, S. , V. Noreika , D. Gueorguiev , Y. Shtyrov , T. A. Bekinschtein , and R. Henson . 2016. “Silent Expectations: Dynamic Causal Modeling of Cortical Prediction and Attention to Sounds That Weren't.” Journal of Neuroscience 36, no. 32: 8305–8316. 27511005 10.1523/JNEUROSCI.1125-16.2016 PMC4978796

[ejn70301-bib-0030] Cowan, N. , I. Winkler , W. Teder , and R. Näätänen . 1993. “Memory Prerequisites of Mismatch Negativity in the Auditory Event‐Related Potential (ERP).” Journal of Experimental Psychology. Learning, Memory, and Cognition 19, no. 4: 909–921. 8345328 10.1037//0278-7393.19.4.909

[ejn70301-bib-1031] Csépe, V. 1995. “On the origin and development of the mismatch negativity.” Ear and hearing 16, no. 1: 91–104. 7774772 10.1097/00003446-199502000-00007

[ejn70301-bib-0031] Czigler, I. , and P. Kojouharova . 2021. “Visual Mismatch Negativity: A Mini‐Review of Non‐pathological Studies With Special Populations and Stimuli.” Frontiers in Human Neuroscience 15: 781234. 35250507 10.3389/fnhum.2021.781234 PMC8888690

[ejn70301-bib-0032] Dayan, P. , and L. F. Abbott . 2001. Theoretical Neuroscience: Computational and Mathematical Modeling of Neural Systems. MIT press.

[ejn70301-bib-0033] de Vignemont, F. , A. Majid , C. Jola , and P. Haggard . 2009. “Segmenting the Body Into Parts: Evidence From Biases in Tactile Perception.” Quarterly Journal of Experimental Psychology (Hove) 62, no. 3: 500–512. 10.1080/17470210802000802 18609376

[ejn70301-bib-0034] Dehaene, S. , F. Meyniel , C. Wacongne , L. Wang , and C. Pallier . 2015. “The Neural Representation of Sequences: From Transition Probabilities to Algebraic Patterns and Linguistic Trees.” Neuron 88, no. 1: 2–19. 26447569 10.1016/j.neuron.2015.09.019

[ejn70301-bib-0035] Delhaye, B. P. , K. H. Long , and S. J. Bensmaia . 2018. “Neural Basis of Touch and Proprioception in Primate Cortex.” Comprehensive Physiology 8, no. 4: 1575–1602. 30215864 10.1002/cphy.c170033 PMC6330897

[ejn70301-bib-0036] Dercksen, T. T. , A. Widmann , T. Noesselt , and N. Wetzel . 2024. “Somatosensory Omissions Reveal Action‐Related Predictive Processing.” Human Brain Mapping 45, no. 4: e26550. 38050773 10.1002/hbm.26550 PMC10915725

[ejn70301-bib-0037] Dercksen, T. T. , A. Widmann , F. Scharf , and N. Wetzel . 2022. “Sound Omission Related Brain Responses in Children.” Developmental Cognitive Neuroscience 53: 101045. 34923314 10.1016/j.dcn.2021.101045 PMC8688889

[ejn70301-bib-0038] Dercksen, T. T. , A. Widmann , E. Schröger , and N. Wetzel . 2020. “Omission Related Brain Responses Reflect Specific and Unspecific Action‐Effect Couplings.” NeuroImage 215: 116840. 32289452 10.1016/j.neuroimage.2020.116840

[ejn70301-bib-0039] Dercksen, T. T. , A. Widmann , and N. Wetzel . 2023. “Salient Omissions‐Pupil Dilation in Response to Unexpected Omissions of Sound and Touch.” Frontiers in Psychiatry 14: 1143931. 37032955 10.3389/fpsyt.2023.1143931 PMC10077953

[ejn70301-bib-0040] Diaz‐Quesada, M. , and M. Maravall . 2008. “Intrinsic Mechanisms for Adaptive Gain Rescaling in Barrel Cortex.” Journal of Neuroscience 28, no. 3: 696–710. 18199769 10.1523/JNEUROSCI.4931-07.2008 PMC6670359

[ejn70301-bib-0041] Dimwamwa, E. D. , A. Pala , V. Chundru , N. C. Wright , and G. B. Stanley . 2024. “Dynamic Corticothalamic Modulation of the Somatosensory Thalamocortical Circuit During Wakefulness.” Nature Communications 15, no. 1: 3529. 10.1038/s41467-024-47863-8 PMC11045850 38664415

[ejn70301-bib-0042] Duerler, P. , S. Brem , G. Fraga‐González , et al. 2021. “Psilocybin Induces Aberrant Prediction Error Processing of Tactile Mismatch Responses‐A Simultaneous EEG‐FMRI Study.” Cerebral Cortex 32, no. 1: 186–196. 34255821 10.1093/cercor/bhab202

[ejn70301-bib-0043] El Karoui, I. , J. R. King , J. Sitt , et al. 2015. “Event‐Related Potential, Time‐Frequency, and Functional Connectivity Facets of Local and Global Auditory Novelty Processing: An Intracranial Study in Humans.” Cerebral Cortex 25, no. 11: 4203–4212. 24969472 10.1093/cercor/bhu143 PMC5635961

[ejn70301-bib-0044] English, G. , N. Ghasemi Nejad , M. Sommerfelt , M. F. Yanik , and W. von der Behrens . 2023. “Bayesian Surprise Shapes Neural Responses in Somatosensory Cortical Circuits.” Cell Reports 42, no. 2: 112009. 36701237 10.1016/j.celrep.2023.112009

[ejn70301-bib-0045] Escera, C. , K. Alho , E. Schröger , and I. W. Winkler . 2000. “Involuntary Attention and Distractibility as Evaluated With Event‐Related Brain Potentials.” Audiology & Neuro‐Otology 5, no. 3–4: 151–166. 10859410 10.1159/000013877

[ejn70301-bib-0046] Estebanez, L. , J. Bertherat , D. E. Shulz , L. Bourdieu , and J. F. Léger . 2016. “A Radial map of Multi‐Whisker Correlation Selectivity in the Rat Barrel Cortex.” Nature Communications 7: 13528. 10.1038/ncomms13528 PMC5121329 27869114

[ejn70301-bib-0047] Fairhall, A. L. , G. D. Lewen , W. Bialek , and R. R. de Ruyter van Steveninck . 2001. “Efficiency and Ambiguity in an Adaptive Neural Code.” Nature 412, no. 6849: 787–792. 11518957 10.1038/35090500

[ejn70301-bib-0048] Farashahi, S. , C. H. Donahue , P. Khorsand , H. Seo , D. Lee , and A. Soltani . 2017. “Metaplasticity as a Neural Substrate for Adaptive Learning and Choice Under Uncertainty.” Neuron 94, no. 2: 401–414 e6. 28426971 10.1016/j.neuron.2017.03.044 PMC5515734

[ejn70301-bib-0049] Fardo, F. , R. Auksztulewicz , M. Allen , M. J. Dietz , A. Roepstorff , and K. J. Friston . 2017. “Expectation Violation and Attention to Pain Jointly Modulate Neural Gain in Somatosensory Cortex.” NeuroImage 153: 109–121. 28341164 10.1016/j.neuroimage.2017.03.041 PMC5460976

[ejn70301-bib-0050] Farley, B. J. , M. C. Quirk , J. J. Doherty , and E. P. Christian . 2010. “Stimulus‐Specific Adaptation in Auditory Cortex Is an NMDA‐Independent Process Distinct From the Sensory Novelty Encoded by the Mismatch Negativity.” Journal of Neuroscience 30, no. 49: 16475–16484. 21147987 10.1523/JNEUROSCI.2793-10.2010 PMC6634869

[ejn70301-bib-0051] Fishman, Y. I. 2014. “The Mechanisms and Meaning of the Mismatch Negativity.” Brain Topography 27, no. 4: 500–526. 24276221 10.1007/s10548-013-0337-3

[ejn70301-bib-0052] Fitzgerald, K. , and J. Todd . 2020. “Making Sense of Mismatch Negativity.” Frontiers in Psychiatry 11: 468. 32595529 10.3389/fpsyt.2020.00468 PMC7300203

[ejn70301-bib-0053] Friedman, D. , Y. M. Cycowicz , and H. Gaeta . 2001. “The Novelty P3: An Event‐Related Brain Potential (ERP) Sign of the Brain's Evaluation of Novelty.” Neuroscience and Biobehavioral Reviews 25, no. 4: 355–373. 11445140 10.1016/s0149-7634(01)00019-7

[ejn70301-bib-0054] Friedman, R. M. , L. M. Chen , and A. W. Roe . 2004. “Modality Maps Within Primate Somatosensory Cortex.” Proceedings of the National Academy of Sciences of the United States of America 101, no. 34: 12724–12729. 15308779 10.1073/pnas.0404884101 PMC514661

[ejn70301-bib-0055] Friston, K. 2005. “A Theory of Cortical Responses.” Philosophical Transactions of the Royal Society of London. Series B, Biological Sciences 360, no. 1456: 815–836. 15937014 10.1098/rstb.2005.1622 PMC1569488

[ejn70301-bib-0056] Friston, K. J. 2019. “Waves of Prediction.” PLoS Biology 17, no. 10: e3000426. 31581195 10.1371/journal.pbio.3000426 PMC6776254

[ejn70301-bib-0057] Ganmor, E. , Y. Katz , and I. Lampl . 2010. “Intensity‐Dependent Adaptation of Cortical and Thalamic Neurons Is Controlled by Brainstem Circuits of the Sensory Pathway.” Neuron 66, no. 2: 273–286. 20435003 10.1016/j.neuron.2010.03.032

[ejn70301-bib-0058] Garrido, M. I. , J. M. Kilner , S. J. Kiebel , and K. J. Friston . 2009. “Dynamic Causal Modeling of the Response to Frequency Deviants.” Journal of Neurophysiology 101, no. 5: 2620–2631. 19261714 10.1152/jn.90291.2008 PMC2681422

[ejn70301-bib-0059] Garrido, M. I. , J. M. Kilner , K. E. Stephan , and K. J. Friston . 2009. “The Mismatch Negativity: A Review of Underlying Mechanisms.” Clinical Neurophysiology 120, no. 3: 453–463. 19181570 10.1016/j.clinph.2008.11.029 PMC2671031

[ejn70301-bib-0060] Garrido, M. I. , M. Sahani , and R. J. Dolan . 2013. “Outlier Responses Reflect Sensitivity to Statistical Structure in the Human Brain.” PLoS Computational Biology 9, no. 3: e1002999. 23555230 10.1371/journal.pcbi.1002999 PMC3610625

[ejn70301-bib-0061] Ghasemi Nejad, N. , G. English , A. Apostolelli , N. Kopp , M. F. Yanik , and W. von der Behrens . 2023. “Deviance Distraction and Stimulus‐Specific Adaptation in the Somatosensory Cortex Reduce With Experience.” Journal of Neuroscience 43, no. 24: 4418–4433. 37169591 10.1523/JNEUROSCI.1714-22.2023 PMC10278680

[ejn70301-bib-0062] Gijsen, S. , M. Grundei , R. T. Lange , D. Ostwald , and F. Blankenburg . 2021. “Neural Surprise in Somatosensory Bayesian Learning.” PLoS Computational Biology 17, no. 2: e1008068. 33529181 10.1371/journal.pcbi.1008068 PMC7880500

[ejn70301-bib-0063] Glaze, C. M. , J. W. Kable , and J. I. Gold . 2015. “Normative Evidence Accumulation in Unpredictable Environments.” eLife 4: e08825. 26322383 10.7554/eLife.08825 PMC4584511

[ejn70301-bib-0064] Grundei, M. , T. T. Schmidt , and F. Blankenburg . 2023. “A Multimodal Cortical Network of Sensory Expectation Violation Revealed by fMRI.” Human Brain Mapping 44, no. 17: 5871–5891. 37721377 10.1002/hbm.26482 PMC10619418

[ejn70301-bib-0065] Grundei, M. , P. Schröder , S. Gijsen , and F. Blankenburg . 2023. “EEG Mismatch Responses in a Multimodal Roving Stimulus Paradigm Provide Evidence for Probabilistic Inference Across Audition, Somatosensation, and Vision.” Human Brain Mapping 44, no. 9: 3644–3668. 37067073 10.1002/hbm.26303 PMC10203815

[ejn70301-bib-0066] Gutlin, D. C. , H. H. McDermott , M. Grundei , and R. Auksztulewicz . 2024. “Model‐Based Approaches to Investigating Mismatch Responses in Schizophrenia.” Clinical EEG and Neuroscience 56: 15500594241253910. 10.1177/15500594241253910 PMC11664892 38751125

[ejn70301-bib-0067] Hamm, J. P. , and R. Yuste . 2016. “Somatostatin Interneurons Control a Key Component of Mismatch Negativity in Mouse Visual Cortex.” Cell Reports 16, no. 3: 597–604. 27396334 10.1016/j.celrep.2016.06.037 PMC4956574

[ejn70301-bib-0068] Han, C. , G. English , H. P. Saal , et al. 2023. “Modelling Novelty Detection in the Thalamocortical Loop.” PLoS Computational Biology 19, no. 5: e1009616. 37186588 10.1371/journal.pcbi.1009616 PMC10212104

[ejn70301-bib-0069] Harms, L. , W. R. Fulham , J. Todd , et al. 2014. “Mismatch Negativity (MMN) in Freely‐Moving Rats With Several Experimental Controls.” PLoS ONE 9, no. 10: e110892. 25333698 10.1371/journal.pone.0110892 PMC4205004

[ejn70301-bib-0070] Harms, L. , P. T. Michie , and R. Naatanen . 2016. “Criteria for Determining Whether Mismatch Responses Exist in Animal Models: Focus on Rodents.” Biological Psychology 116: 28–35. 26196895 10.1016/j.biopsycho.2015.07.006

[ejn70301-bib-0071] Hautasaari, P. , U. M. Kujala , and I. M. Tarkka . 2019. “Detecting Differences With Magnetoencephalography of Somatosensory Processing After Tactile and Electrical Stimuli.” Journal of Neuroscience Methods 311: 331–337. 30218670 10.1016/j.jneumeth.2018.09.014

[ejn70301-bib-0072] Hautasaari, P. , A. M. Savić , O. Loberg , et al. 2017. “Somatosensory Brain Function and Gray Matter Regional Volumes Differ According to Exercise History: Evidence From Monozygotic Twins.” Brain Topography 30, no. 1: 77–86. 27761665 10.1007/s10548-016-0531-1

[ejn70301-bib-0073] He, X. , J. Zhang , Z. Zhang , et al. 2020. “Effects of Visual Attentional Load on the Tactile Sensory Memory Indexed by Somatosensory Mismatch Negativity.” Frontiers in Neuroinformatics 14: 575078. 33324187 10.3389/fninf.2020.575078 PMC7724049

[ejn70301-bib-0074] Heilbron, M. , and M. Chait . 2018. “Great Expectations: Is There Evidence for Predictive Coding in Auditory Cortex?” Neuroscience 389: 54–73. 28782642 10.1016/j.neuroscience.2017.07.061

[ejn70301-bib-0075] Horvath, J. , and I. Winkler . 2004. “How the Human Auditory System Treats Repetition Amongst Change.” Neuroscience Letters 368, no. 2: 157–161. 15351440 10.1016/j.neulet.2004.07.004

[ejn70301-bib-0076] Hu, L. , C. Zhao , H. Li , and E. Valentini . 2013. “Mismatch Responses Evoked by Nociceptive Stimuli.” Psychophysiology 50, no. 2: 158–173. 23256883 10.1111/psyp.12000

[ejn70301-bib-0077] Huang, M. X. , R. R. Lee , G. A. Miller , et al. 2005. “A Parietal‐Frontal Network Studied by Somatosensory Oddball MEG Responses, and Its Cross‐Modal Consistency.” NeuroImage 28, no. 1: 99–114. 15979344 10.1016/j.neuroimage.2005.05.036

[ejn70301-bib-0078] Hughes, H. C. , T. M. Darcey , H. I. Barkan , P. D. Williamson , D. W. Roberts , and C. H. Aslin . 2001. “Responses of Human Auditory Association Cortex to the Omission of an Expected Acoustic Event.” Neuroimage 13, no. 6 Pt 1: 1073–1089. 11352613 10.1006/nimg.2001.0766

[ejn70301-bib-0079] Isenstein, E. L. , E. G. Freedman , S. Molholm , and J. J. Foxe . 2024. “Somatosensory Temporal Sensitivity in Adults on the Autism Spectrum: A High‐Density Electrophysiological Mapping Study Using the Mismatch Negativity (MMN) Sensory Memory Paradigm.” Autism Research 17, no. 9: 1760–1777. 38973746 10.1002/aur.3186

[ejn70301-bib-0080] Isenstein, E. L. , E. G. Freedman , G. A. Rico , Z. Brown , D. Tadin , and J. J. Foxe . 2025. “Adults on the Autism Spectrum Differ From Neurotypical Peers When Self‐Generating but Not Passively‐Experiencing Somatosensation: A High‐Density Electrophysiological (EEG) Mapping and Virtual Reality Study.” NeuroImage 311: 121215. 40228683 10.1016/j.neuroimage.2025.121215 PMC12173217

[ejn70301-bib-0081] Isenstein, E. L. , E. G. Freedman , A. J. Xu , I. A. DeAndrea‐Lazarus , and J. J. Foxe . 2024. “Probing the Neurophysiology of Temporal Sensitivity in the Somatosensory System Using the Mismatch Negativity (MMN) Sensory Memory Paradigm.” Neuroscience 536: 47–56. 37979841 10.1016/j.neuroscience.2023.11.013 PMC11008681

[ejn70301-bib-0082] Iwamura, Y. 1998. “Hierarchical Somatosensory Processing.” Current Opinion in Neurobiology 8, no. 4: 522–528. 9751655 10.1016/s0959-4388(98)80041-x

[ejn70301-bib-0083] Jääskelainen, I. P. , J. Ahveninen , G. Bonmassar , et al. 2004. “Human Posterior Auditory Cortex Gates Novel Sounds to Consciousness.” Proceedings of the National Academy of Sciences of the United States of America 101, no. 17: 6809–6814. 15096618 10.1073/pnas.0303760101 PMC404127

[ejn70301-bib-0084] Jacobsen, T. , and E. Schroger . 2001. “Is There pre‐Attentive Memory‐Based Comparison of Pitch?” Psychophysiology 38, no. 4: 723–727. 11446587

[ejn70301-bib-1085] Johansson, R. S. , and J. R. Flanagan . 2008. Tactile Sensory Control of Object Manipulation in Humans, in The Senses: A Comprehensive Reference, Vol. 6, pp. 67–86.

[ejn70301-bib-0085] Jordan, R. , and G. B. Keller . 2023. “The Locus Coeruleus Broadcasts Prediction Errors Across the Cortex to Promote Sensorimotor Plasticity.” eLife 12: RP85111. 37285281 10.7554/eLife.85111 PMC10328511

[ejn70301-bib-0086] Jubran, M. , B. Mohar , and I. Lampl . 2016. “The Transformation of Adaptation Specificity to Whisker Identity From Brainstem to Thalamus.” Frontiers in Systems Neuroscience 10: 56. 27445716 10.3389/fnsys.2016.00056 PMC4917531

[ejn70301-bib-0087] Kaas, J. H. 1993. “The Functional Organization of Somatosensory Cortex in primates.” Annals of Anatomy 175, no. 6: 509–518. 8297039 10.1016/s0940-9602(11)80212-8

[ejn70301-bib-0088] Kangas, E. S. , X. Li , E. Vuoriainen , S. Lindeman , and P. Astikainen . 2024. “Intensity Dependence of Auditory Evoked Potentials Distinguish Participants With Unmedicated Depression From Non‐Depressed Controls.” European Journal of Neuroscience 60, no. 10: 6440–6469. 39401940 10.1111/ejn.16569

[ejn70301-bib-0089] Katz, Y. , J. E. Heiss , and I. Lampl . 2006. “Cross‐Whisker Adaptation of Neurons in the Rat Barrel Cortex.” Journal of Neuroscience 26, no. 51: 13363–13372. 17182787 10.1523/JNEUROSCI.4056-06.2006 PMC6674994

[ejn70301-bib-0090] Kekoni, J. , H. Hämäläinen , M. Saarinen , et al. 1997. “Rate Effect and Mismatch Responses in the Somatosensory System: ERP‐Recordings in Humans.” Biological Psychology 46, no. 2: 125–142. 9288410 10.1016/s0301-0511(97)05249-6

[ejn70301-bib-0091] Keller, G. B. , T. Bonhoeffer , and M. Hubener . 2012. “Sensorimotor Mismatch Signals in Primary Visual Cortex of the Behaving Mouse.” Neuron 74, no. 5: 809–815. 22681686 10.1016/j.neuron.2012.03.040

[ejn70301-bib-0092] Keller, G. B. , and T. D. Mrsic‐Flogel . 2018. “Predictive Processing: A Canonical Cortical Computation.” Neuron 100, no. 2: 424–435. 30359606 10.1016/j.neuron.2018.10.003 PMC6400266

[ejn70301-bib-0093] Khouri, L. , and I. Nelken . 2015. “Detecting the Unexpected.” Current Opinion in Neurobiology 35: 142–147. 26318534 10.1016/j.conb.2015.08.003

[ejn70301-bib-0094] Knill, D. C. , and A. Pouget . 2004. “The Bayesian Brain: The Role of Uncertainty in Neural Coding and Computation.” Trends in Neurosciences 27, no. 12: 712–719. 15541511 10.1016/j.tins.2004.10.007

[ejn70301-bib-0095] Lampl, I. , and Y. Katz . 2017. “Neuronal Adaptation in the Somatosensory System of Rodents.” Neuroscience 343: 66–76. 27923742 10.1016/j.neuroscience.2016.11.043

[ejn70301-bib-0096] Lao‐Rodriguez, A. B. , K. Przewrocki , D. Pérez‐González , et al. 2023. “Neuronal Responses to Omitted Tones in the Auditory Brain: A Neuronal Correlate for Predictive Coding.” Science Advances 9, no. 24: eabq8657. 37315139 10.1126/sciadv.abq8657 PMC10266733

[ejn70301-bib-0097] Lecaignard, F. , O. Bertrand , A. Caclin , and J. Mattout . 2022. “Neurocomputational Underpinnings of Expected Surprise.” Journal of Neuroscience 42, no. 3: 474–486. 34819342 10.1523/JNEUROSCI.0601-21.2021 PMC8802931

[ejn70301-bib-0098] Lecaignard, F. , R. Bertrand , P. Brunner , A. Caclin , G. Schalk , and J. Mattout . 2021. “Dynamics of Oddball Sound Processing: Trial‐By‐Trial Modeling of ECoG Signals.” Frontiers in Human Neuroscience 15: 794654. 35221952 10.3389/fnhum.2021.794654 PMC8866734

[ejn70301-bib-0099] Lee, S. , G. E. Carvell , and D. J. Simons . 2008. “Motor Modulation of Afferent Somatosensory Circuits.” Nature Neuroscience 11, no. 12: 1430–1438. 19011625 10.1038/nn.2227 PMC2597103

[ejn70301-bib-0100] Lieder, F. , J. Daunizeau , M. I. Garrido , K. J. Friston , and K. E. Stephan . 2013. “Modelling Trial‐By‐Trial Changes in the Mismatch Negativity.” PLoS Computational Biology 9, no. 2: e1002911. 23436989 10.1371/journal.pcbi.1002911 PMC3578779

[ejn70301-bib-0101] Liu, C. , G. Foffani , A. Scaglione , J. Aguilar , and K. A. Moxon . 2017. “Adaptation of Thalamic Neurons Provides Information About the Spatiotemporal Context of Stimulus History.” Journal of Neuroscience 37, no. 41: 10012–10021. 28899918 10.1523/JNEUROSCI.0637-17.2017 PMC5637111

[ejn70301-bib-0102] Macdonald, M. , and K. Campbell . 2011. “Effects of a Violation of an Expected Increase or Decrease in Intensity on Detection of Change Within an Auditory Pattern.” Brain and Cognition 77, no. 3: 438–445. 21925782 10.1016/j.bandc.2011.08.014

[ejn70301-bib-0103] Maheu, M. , S. Dehaene , and F. Meyniel . 2019. “Brain Signatures of a Multiscale Process of Sequence Learning in Humans.” eLife 8: e41541. 30714904 10.7554/eLife.41541 PMC6361584

[ejn70301-bib-0104] Maravall, M. , R. S. Petersen , A. L. Fairhall , E. Arabzadeh , and M. E. Diamond . 2007. “Shifts in Coding Properties and Maintenance of Information Transmission During Adaptation in Barrel Cortex.” PLoS Biology 5, no. 2: e19. 17253902 10.1371/journal.pbio.0050019 PMC1779810

[ejn70301-bib-0107] May, P. J. C. 2021. “The Adaptation Model Offers a Challenge for the Predictive Coding Account of Mismatch Negativity.” Frontiers in Human Neuroscience 15: 721574. 34867238 10.3389/fnhum.2021.721574 PMC8640521

[ejn70301-bib-0106] May, P. J. , and H. Tiitinen . 2010. “Mismatch Negativity (MMN), the Deviance‐Elicited Auditory Deflection, Explained.” Psychophysiology 47, no. 1: 66–122. 19686538 10.1111/j.1469-8986.2009.00856.x

[ejn70301-bib-0105] May, P. , H. Tiitinen , R. J. Ilmoniemi , G. Nyman , J. G. Taylor , and R. Näätänen . 1999. “Frequency Change Detection in Human Auditory Cortex.” Journal of Computational Neuroscience 6, no. 2: 99–120. 10333158 10.1023/a:1008896417606

[ejn70301-bib-0108] Mease, R. A. , P. Krieger , and A. Groh . 2014. “Cortical Control of Adaptation and Sensory Relay Mode in the Thalamus.” Proceedings of the National Academy of Sciences of the United States of America 111, no. 18: 6798–6803. 24748112 10.1073/pnas.1318665111 PMC4020068

[ejn70301-bib-0109] Meyniel, F. 2020. “Brain Dynamics for Confidence‐Weighted Learning.” PLoS Computational Biology 16, no. 6: e1007935. 32484806 10.1371/journal.pcbi.1007935 PMC7292419

[ejn70301-bib-0110] Meyniel, F. , and S. Dehaene . 2017. “Brain Networks for Confidence Weighting and Hierarchical Inference During Probabilistic Learning.” Proceedings of the National Academy of Sciences of the United States of America 114, no. 19: E3859–E3868. 28439014 10.1073/pnas.1615773114 PMC5441722

[ejn70301-bib-0111] Meyniel, F. , M. Maheu , and S. Dehaene . 2016. “Human Inferences About Sequences: A Minimal Transition Probability Model.” PLoS Computational Biology 12, no. 12: e1005260. 28030543 10.1371/journal.pcbi.1005260 PMC5193331

[ejn70301-bib-0112] Michie, P. T. , M. S. Malmierca , L. Harms , and J. Todd . 2016. “The Neurobiology of MMN and Implications for Schizophrenia.” Biological Psychology 116: 90–97. 26826620 10.1016/j.biopsycho.2016.01.011

[ejn70301-bib-0113] Modirshanechi, A. , J. Brea , and W. Gerstner . 2022. “A Taxonomy of Surprise Definitions.” Journal of Mathematical Psychology 110: 102712.

[ejn70301-bib-0114] Modirshanechi, A. , M. M. Kiani , and H. Aghajan . 2019. “Trial‐By‐Trial Surprise‐Decoding Model for Visual and Auditory Binary Oddball Tasks.” NeuroImage 196: 302–317. 30980899 10.1016/j.neuroimage.2019.04.028

[ejn70301-bib-0115] Mohar, B. , E. Ganmor , and I. Lampl . 2015. “Faithful Representation of Tactile Intensity Under Different Contexts Emerges From the Distinct Adaptive Properties of the First Somatosensory Relay Stations.” Journal of Neuroscience 35, no. 18: 6997–7002. 25948252 10.1523/JNEUROSCI.4358-14.2015 PMC6605262

[ejn70301-bib-0116] Mulders, D. , B. Seymour , A. Mouraux , and F. Mancini . 2023. “Confidence of Probabilistic Predictions Modulates the Cortical Response to Pain.” Proceedings of the National Academy of Sciences of the United States of America 120, no. 4: e2212252120. 36669115 10.1073/pnas.2212252120 PMC9942789

[ejn70301-bib-0117] Musall, S. , F. Haiss , B. Weber , and W. von der Behrens . 2017. “Deviant Processing in the Primary Somatosensory Cortex.” Cerebral Cortex 27, no. 1: 863–876. 26628563 10.1093/cercor/bhv283

[ejn70301-bib-0118] Näätänen, R. 2009. “Somatosensory Mismatch Negativity: A New Clinical Tool for Developmental Neurological Research?” Developmental Medicine and Child Neurology 51, no. 12: 930–931. 19909306 10.1111/j.1469-8749.2009.03386.x

[ejn70301-bib-0119] Näätänen, R. , P. Astikainen , T. Ruusuvirta , and M. Huotilainen . 2010. “Automatic Auditory Intelligence: An Expression of the Sensory‐Cognitive Core of Cognitive Processes.” Brain Research Reviews 64, no. 1: 123–136. 20298716 10.1016/j.brainresrev.2010.03.001

[ejn70301-bib-0120] Näätänen, R. , A. W. Gaillard , and S. Mäntysalo . 1978. “Early Selective‐Attention Effect on Evoked Potential Reinterpreted.” Acta Psychologica 42, no. 4: 313–329. 685709 10.1016/0001-6918(78)90006-9

[ejn70301-bib-0121] Näätänen, R. , T. Jacobsen , and I. Winkler . 2005. “Memory‐Based or Afferent Processes in Mismatch Negativity (MMN): A Review of the Evidence.” Psychophysiology 42, no. 1: 25–32. 15720578 10.1111/j.1469-8986.2005.00256.x

[ejn70301-bib-0122] Näätänen, R. , T. Kujala , and G. Light . 2019. Mismatch Negativity: A Window to the Brain. Oxford University Press.

[ejn70301-bib-0123] Näätänen, R. , E. Schröger , S. Karakas , M. Tervaniemi , and P. Paavilainen . 1993. “Development of a Memory Trace for a Complex Sound in the Human Brain.” Neuroreport 4, no. 5: 503–506. 8513127 10.1097/00001756-199305000-00010

[ejn70301-bib-0124] Näätänen, R. , M. Tervaniemi , E. Sussman , P. Paavilainen , and I. Winkler . 2001. ““Primitive Intelligence” in the Auditory Cortex.” Trends in Neurosciences 24, no. 5: 283–288. 11311381 10.1016/s0166-2236(00)01790-2

[ejn70301-bib-0125] Naeije, G. , T. Vaulet , V. Wens , B. Marty , S. Goldman , and X. De Tiège . 2016. “Multilevel Cortical Processing of Somatosensory Novelty: A Magnetoencephalography Study.” Frontiers in Human Neuroscience 10: 259. 27313523 10.3389/fnhum.2016.00259 PMC4889577

[ejn70301-bib-0126] Naeije, G. , T. Vaulet , V. Wens , B. Marty , S. Goldman , and X. de Tiège . 2018. “Neural Basis of Early Somatosensory Change Detection: A Magnetoencephalography Study.” Brain Topography 31, no. 2: 242–256. 28913778 10.1007/s10548-017-0591-x

[ejn70301-bib-0127] Nelken, I. , and N. Ulanovsky . 2007. “Mismatch Negativity and Stimulus‐Specific Adaptation in Animal Models.” Journal of Psychophysiology 21, no. 3–4: 214–223.

[ejn70301-bib-0128] Niedernhuber, M. , F. Raimondo , J. D. Sitt , and T. A. Bekinschtein . 2022. “Sensory Target Detection at Local and Global Timescales Reveals a Hierarchy of Supramodal Dynamics in the Human Cortex.” Journal of Neuroscience 42: 8729–8741. 36223999 10.1523/JNEUROSCI.0658-22.2022 PMC9671580

[ejn70301-bib-1129] Nieto‐Diego, J. , and M. S. Malmierca . 2016. “Topographic distribution of stimulus‐specific adaptation across auditory cortical fields in the anesthetized rat.” PLoS biology 14, no. 3: e1002397. 26950883 10.1371/journal.pbio.1002397 PMC4780834

[ejn70301-bib-0129] Nordby, H. , W. T. Roth , and A. Pfefferbaum . 1988. “Event‐Related Potentials to Breaks in Sequences of Alternating Pitches or Interstimulus Intervals.” Psychophysiology 25, no. 3: 262–268. 3406327 10.1111/j.1469-8986.1988.tb01239.x

[ejn70301-bib-0130] Olsen, S. R. , D. S. Bortone , H. Adesnik , and M. Scanziani . 2012. “Gain Control by Layer Six in Cortical Circuits of Vision.” Nature 483, no. 7387: 47–52. 22367547 10.1038/nature10835 PMC3636977

[ejn70301-bib-0131] Ostwald, D. , B. Spitzer , M. Guggenmos , T. T. Schmidt , S. J. Kiebel , and F. Blankenburg . 2012. “Evidence for Neural Encoding of Bayesian Surprise in Human Somatosensation.” NeuroImage 62, no. 1: 177–188. 22579866 10.1016/j.neuroimage.2012.04.050

[ejn70301-bib-0132] Paavilainen, P. 2013. “The Mismatch‐Negativity (MMN) Component of the Auditory Event‐Related Potential to Violations of Abstract Regularities: A Review.” International Journal of Psychophysiology 88, no. 2: 109–123. 23542165 10.1016/j.ijpsycho.2013.03.015

[ejn70301-bib-0133] Paavilainen, P. , P. Arajarvi , and R. Takegata . 2007. “Preattentive Detection of Nonsalient Contingencies Between Auditory Features.” Neuroreport 18, no. 2: 159–163. 17301682 10.1097/WNR.0b013e328010e2ac

[ejn70301-bib-0134] Paavilainen, P. , M. Jaramillo , R. Näätänen , and I. Winkler . 1999. “Neuronal Populations in the Human Brain Extracting Invariant Relationships From Acoustic Variance.” Neuroscience Letters 265, no. 3: 179–182. 10327160 10.1016/s0304-3940(99)00237-2

[ejn70301-bib-0135] Penfield, W. , and E. Boldrey . 1937. “Somatic Motor and Sensory Representation in the Cerebral Cortex of Man as Studied by Electrical Stimulation.” Brain 60, no. 4: 389–443.

[ejn70301-bib-0136] Pesonen, H. , J. Strömmer , X. Li , J. Parkkari , I. M. Tarkka , and P. Astikainen . 2023. “Magnetoencephalography Reveals Impaired Sensory Gating and Change Detection in Older Adults in the Somatosensory System.” Neuropsychologia 190: 108702. 37838067 10.1016/j.neuropsychologia.2023.108702

[ejn70301-bib-0137] Petersen, C. C. 2007. “The Functional Organization of the Barrel Cortex.” Neuron 56, no. 2: 339–355. 17964250 10.1016/j.neuron.2007.09.017

[ejn70301-bib-0138] Petersen, C. C. H. 2019. “Sensorimotor Processing in the Rodent Barrel Cortex.” Nature Reviews. Neuroscience 20, no. 9: 533–546. 31367018 10.1038/s41583-019-0200-y PMC7116865

[ejn70301-bib-0139] Polich, J. 2007. “Updating P300: An Integrative Theory of P3a and P3b.” Clinical Neurophysiology 118, no. 10: 2128–2148. 17573239 10.1016/j.clinph.2007.04.019 PMC2715154

[ejn70301-bib-0140] Poublan‐Couzardot, A. , F. Lecaignard , E. Fucci , et al. 2023. “Time‐Resolved Dynamic Computational Modeling of Human EEG Recordings Reveals Gradients of Generative Mechanisms for the MMN Response.” PLoS Computational Biology 19, no. 12: e1010557. 10.1371/journal.pcbi.1010557. 38091350 PMC10752554

[ejn70301-bib-0141] Prete, D. A. , D. Heikoop , J. E. McGillivray , J. P. Reilly , and L. J. Trainor . 2022. “The Sound of Silence: Predictive Error Responses to Unexpected Sound Omission in Adults.” European Journal of Neuroscience 55: 1972–1985. 35357048 10.1111/ejn.15660

[ejn70301-bib-0142] Restuccia, D. , G. Della Marca , M. Valeriani , M. G. Leggio , and M. Molinari . 2007. “Cerebellar Damage Impairs Detection of Somatosensory Input Changes. *A Somatosensory Mismatch‐Negativity Study* .” Brain 130, no. Pt 1: 276–287. 16982654 10.1093/brain/awl236

[ejn70301-bib-0143] Restuccia, D. , S. Zanini , M. Cazzagon , I. Del Piero , L. Martucci , and G. Della Marca . 2009. “Somatosensory Mismatch Negativity in Healthy Children.” Developmental Medicine and Child Neurology 51, no. 12: 991–998. 19909309 10.1111/j.1469-8749.2009.03367.x

[ejn70301-bib-0144] Ritter, W. , P. Paavilainen , J. Lavikainen , et al. 1992. “Event‐Related Potentials to Repetition and Change of Auditory Stimuli.” Electroencephalography and Clinical Neurophysiology 83, no. 5: 306–321. 1385087 10.1016/0013-4694(92)90090-5

[ejn70301-bib-0145] Ross, J. M. , and J. P. Hamm . 2020. “Cortical Microcircuit Mechanisms of Mismatch Negativity and Its Underlying Subcomponents.” Frontiers in Neural Circuits 14: 13. 32296311 10.3389/fncir.2020.00013 PMC7137737

[ejn70301-bib-0146] Ruhnau, P. , B. Herrmann , and E. Schroger . 2012. “Finding the Right Control: The Mismatch Negativity Under Investigation.” Clinical Neurophysiology 123, no. 3: 507–512. 21839676 10.1016/j.clinph.2011.07.035

[ejn70301-bib-0147] Ruusuvirta, T. , K. Koivisto , J. Wikgren , and P. Astikainen . 2007. “Processing of Melodic Contours in Urethane‐Anaesthetized Rats.” European Journal of Neuroscience 26, no. 3: 701–703. 17634069 10.1111/j.1460-9568.2007.05687.x

[ejn70301-bib-0148] Ryali, C. K. , G. Reddy , and A. J. Yu . 2018. “Demystifying Excessively Volatile Human Learning: A Bayesian Persistent Prior and a Neural Approximation.” Advances in Neural Information Processing Systems 31: 2781–2790. 34366637 PMC8341474

[ejn70301-bib-0149] Saarinen, J. , P. Paavilainen , E. Schöger , M. Tervaniemi , and R. Näätänen . 1992. “Representation of Abstract Attributes of Auditory Stimuli in the Human Brain.” Neuroreport 3, no. 12: 1149–1151. 1493229 10.1097/00001756-199212000-00030

[ejn70301-bib-0150] Salisbury, D. F. 2012. “Finding the Missing Stimulus Mismatch Negativity (MMN): Emitted MMN to Violations of an Auditory Gestalt.” Psychophysiology 49, no. 4: 544–548. 22221004 10.1111/j.1469-8986.2011.01336.x PMC3309149

[ejn70301-bib-0151] SanMiguel, I. , K. Saupe , and E. Schröger . 2013. “I Know What Is Missing here: Electrophysiological Prediction Error Signals Elicited by Omissions of Predicted “What” but Not “When”.” Frontiers in Human Neuroscience 7: 407. 23908618 10.3389/fnhum.2013.00407 PMC3725431

[ejn70301-bib-0152] SanMiguel, I. , A. Widmann , A. Bendixen , N. Trujillo‐Barreto , and E. Schröger . 2013. “Hearing Silences: Human Auditory Processing Relies on Preactivation of Sound‐Specific Brain Activity Patterns.” Journal of Neuroscience 33, no. 20: 8633–8639. 23678108 10.1523/JNEUROSCI.5821-12.2013 PMC6618825

[ejn70301-bib-0153] Schlossmacher, I. , F. Lucka , A. Peters , M. Bruchmann , and T. Straube . 2022. “Effects of Awareness and Task Relevance on Neurocomputational Models of Mismatch Negativity Generation.” NeuroImage 262: 119530. 35940422 10.1016/j.neuroimage.2022.119530

[ejn70301-bib-0154] Schröger, E. , U. Roeber , and N. Coy . 2023. “Markov Chains as a Proxy for the Predictive Memory Representations Underlying Mismatch Negativity.” Frontiers in Human Neuroscience 17: 1249413. 37771348 10.3389/fnhum.2023.1249413 PMC10525344

[ejn70301-bib-0155] Schroger, E. , and C. Wolff . 1996. “Mismatch Response of the Human Brain to Changes in Sound Location.” Neuroreport 7, no. 18: 3005–3008. 9116228 10.1097/00001756-199611250-00041

[ejn70301-bib-0156] Shen, G. , A. N. Meltzoff , S. M. Weiss , and P. J. Marshall . 2020. “Body Representation in Infants: Categorical Boundaries of Body Parts as Assessed by Somatosensory Mismatch Negativity.” Developmental Cognitive Neuroscience 44: 100795. 32716850 10.1016/j.dcn.2020.100795 PMC7303979

[ejn70301-bib-0157] Shen, G. , N. J. Smyk , A. N. Meltzoff , and P. J. Marshall . 2018a. “Using Somatosensory Mismatch Responses as a Window Into Somatotopic Processing of Tactile Stimulation.” Psychophysiology 55, no. 5: e13030. 29139557 10.1111/psyp.13030

[ejn70301-bib-0158] Shen, G. , N. J. Smyk , A. N. Meltzoff , and P. J. Marshall . 2018b. “Neuropsychology of Human Body Parts: Exploring Categorical Boundaries of Tactile Perception Using Somatosensory Mismatch Responses.” Journal of Cognitive Neuroscience 30, no. 12: 1858–1869. 30024330 10.1162/jocn_a_01313 PMC12884974

[ejn70301-bib-0159] Shinozaki, N. , H. Yabe , T. Sutoh , T. Hiruma , and S. Kaneko . 1998. “Somatosensory Automatic Responses to Deviant Stimuli.” Brain Research. Cognitive Brain Research 7, no. 2: 165–171. 9774724 10.1016/s0926-6410(98)00020-2

[ejn70301-bib-0160] Shiramatsu, T. I. , and H. Takahashi . 2021. “Mismatch‐Negativity (MMN) in Animal Models: Homology of Human MMN?” Hearing Research 399: 107936. 32197715 10.1016/j.heares.2020.107936

[ejn70301-bib-0161] Shymkiv, Y. , J. P. Hamm , S. Escola , and R. Yuste . 2025. “Slow Cortical Dynamics Generate Context Processing and Novelty Detection.” Neuron 113: 847–857.e8. 39933524 10.1016/j.neuron.2025.01.011 PMC11925667

[ejn70301-bib-0162] Smirnakis, S. M. , M. J. Berry , D. K. Warland , W. Bialek , and M. Meister . 1997. “Adaptation of Retinal Processing to Image Contrast and Spatial Scale.” Nature 386, no. 6620: 69–73. 9052781 10.1038/386069a0

[ejn70301-bib-0163] Sokolov, E. N. 1963. “Higher Nervous Functions; the Orienting Reflex.” Annual Review of Physiology 25: 545–580. 10.1146/annurev.ph.25.030163.002553 13977960

[ejn70301-bib-0164] Spackman, L. A. , S. G. Boyd , and A. Towell . 2007. “Effects of Stimulus Frequency and Duration on Somatosensory Discrimination Responses.” Experimental Brain Research 177, no. 1: 21–30. 16917766 10.1007/s00221-006-0650-0

[ejn70301-bib-0165] Spackman, L. A. , A. Towell , and S. G. Boyd . 2010. “Somatosensory Discrimination: An Intracranial Event‐Related Potential Study of Children With Refractory Epilepsy.” Brain Research 1310: 68–76. 19896930 10.1016/j.brainres.2009.10.072

[ejn70301-bib-0166] Stefanics, G. , J. Heinzle , A. A. Horváth , and K. E. Stephan . 2018. “Visual Mismatch and Predictive Coding: A Computational Single‐Trial ERP Study.” Journal of Neuroscience 38, no. 16: 4020–4030. 29581379 10.1523/JNEUROSCI.3365-17.2018 PMC6705923

[ejn70301-bib-0167] Sterzer, P. , R. A. Adams , P. Fletcher , et al. 2018. “The Predictive Coding Account of Psychosis.” Biological Psychiatry 84, no. 9: 634–643. 30007575 10.1016/j.biopsych.2018.05.015 PMC6169400

[ejn70301-bib-0168] Strömmer, J. M. , N. Põldver , T. Waselius , et al. 2017. “Automatic Auditory and Somatosensory Brain Responses in Relation to Cognitive Abilities and Physical Fitness in Older Adults.” Scientific Reports 7: 7. 29057924 10.1038/s41598-017-14139-9 PMC5651800

[ejn70301-bib-0169] Strömmer, J. M. , I. M. Tarkka , and P. Astikainen . 2014. “Somatosensory Mismatch Response in Young and Elderly Adults.” Frontiers in Aging Neuroscience 6: 293. 25386140 10.3389/fnagi.2014.00293 PMC4209888

[ejn70301-bib-0170] Taaseh, N. , A. Yaron , and I. Nelken . 2011. “Stimulus‐Specific Adaptation and Deviance Detection in the rat Auditory Cortex.” PLoS ONE 6, no. 8: e23369. 21853120 10.1371/journal.pone.0023369 PMC3154435

[ejn70301-bib-0171] Tervaniemi, M. , S. Maury , and R. Näätänen . 1994. “Neural Representations of Abstract Stimulus Features in the Human Brain as Reflected by the Mismatch Negativity.” Neuroreport 5, no. 7: 844–846. 8018861 10.1097/00001756-199403000-00027

[ejn70301-bib-0172] Thomson, A. M. 2010. “Neocortical Layer 6, A Review.” Frontiers in Neuroanatomy 4: 13. 20556241 10.3389/fnana.2010.00013 PMC2885865

[ejn70301-bib-0173] Todd, J. , D. Salisbury , and P. T. Michie . 2023. “Why Mismatch Negativity Continues to Hold Potential in Probing Altered Brain Function in Schizophrenia.” Psychiatry and Clinical Neurosciences Reports 2, no. 3: e144. 38867817 10.1002/pcn5.144 PMC11114358

[ejn70301-bib-0174] Uhrig, L. , S. Dehaene , and B. Jarraya . 2014. “A Hierarchy of Responses to Auditory Regularities in the Macaque Brain.” Journal of Neuroscience 34, no. 4: 1127–1132. 24453305 10.1523/JNEUROSCI.3165-13.2014 PMC5635960

[ejn70301-bib-0175] Ulanovsky, N. , L. Las , and I. Nelken . 2003. “Processing of Low‐Probability Sounds by Cortical Neurons.” Nature Neuroscience 6, no. 4: 391–398. 12652303 10.1038/nn1032

[ejn70301-bib-0176] Velez‐Fort, M. , C. V. Rousseau , C. J. Niedworok , et al. 2014. “The Stimulus Selectivity and Connectivity of Layer six Principal Cells Reveals Cortical Microcircuits Underlying Visual Processing.” Neuron 83, no. 6: 1431–1443. 25175879 10.1016/j.neuron.2014.08.001 PMC4175007

[ejn70301-bib-0177] Voigts, J. , C. A. Deister , and C. I. Moore . 2020. “Layer 6 Ensembles Can Selectively Regulate the Behavioral Impact and Layer‐Specific Representation of Sensory Deviants.” eLife 9: e48957. 33263283 10.7554/eLife.48957 PMC7817180

[ejn70301-bib-0178] Voigts, J. , B. Sakmann , and T. Celikel . 2008. “Unsupervised Whisker Tracking in Unrestrained Behaving Animals.” Journal of Neurophysiology 100, no. 1: 504–515. 18463190 10.1152/jn.00012.2008

[ejn70301-bib-0179] von der Behrens, W. , P. Bäuerle , M. Kössl , and B. H. Gaese . 2009. “Correlating Stimulus‐Specific Adaptation of Cortical Neurons and Local Field Potentials in the Awake rat.” Journal of Neuroscience 29, no. 44: 13837–13849. 19889995 10.1523/JNEUROSCI.3475-09.2009 PMC6666711

[ejn70301-bib-0180] Wacongne, C. , E. Labyt , V. van Wassenhove , T. Bekinschtein , L. Naccache , and S. Dehaene . 2011. “Evidence for a Hierarchy of Predictions and Prediction Errors in Human Cortex.” Proceedings of the National Academy of Sciences of the United States of America 108, no. 51: 20754–20759. 22147913 10.1073/pnas.1117807108 PMC3251061

[ejn70301-bib-0181] Wark, B. , B. N. Lundstrom , and A. Fairhall . 2007. “Sensory Adaptation.” Current Opinion in Neurobiology 17, no. 4: 423–429. 17714934 10.1016/j.conb.2007.07.001 PMC2084204

[ejn70301-bib-0182] Waters, R. S. , C. X. Li , and C. A. McCandlish . 1995. “Relationship Between the Organization of the Forepaw Barrel Subfield and the Representation of the Forepaw in Layer IV of rat Somatosensory Cortex.” Experimental Brain Research 103, no. 2: 183–197. 7789426 10.1007/BF00231705

[ejn70301-bib-0183] Weber, L. A. , A. O. Diaconescu , C. Mathys , et al. 2020. “Ketamine Affects Prediction Errors About Statistical Regularities: A Computational Single‐Trial Analysis of the Mismatch Negativity.” Journal of Neuroscience 40, no. 29: 5658–5668. 32561673 10.1523/JNEUROSCI.3069-19.2020 PMC7363468

[ejn70301-bib-0184] Wetzel, N. , and E. Schröger . 2014. “On the Development of Auditory Distraction: A Review.” PsyCh Journal 3, no. 1: 72–91. 26271640 10.1002/pchj.49

[ejn70301-bib-0185] Whitmire, C. J. , and G. B. Stanley . 2016. “Rapid Sensory Adaptation Redux: A Circuit Perspective.” Neuron 92, no. 2: 298–315. 27764664 10.1016/j.neuron.2016.09.046 PMC5076890

[ejn70301-bib-0186] Winkler, I. 2007. “Interpreting the Mismatch Negativity.” Journal of Psychophysiology 21, no. 3–4: 147–163.

[ejn70301-bib-0187] Winkler, I. , and I. Czigler . 2012. “Evidence From Auditory and Visual Event‐Related Potential (ERP) Studies of Deviance Detection (MMN and vMMN) Linking Predictive Coding Theories and Perceptual Object Representations.” International Journal of Psychophysiology 83, no. 2: 132–143. 22047947 10.1016/j.ijpsycho.2011.10.001

[ejn70301-bib-0188] Winkler, I. , S. L. Denham , and I. Nelken . 2009. “Modeling the Auditory Scene: Predictive Regularity Representations and Perceptual Objects.” Trends in Cognitive Sciences 13, no. 12: 532–540. 19828357 10.1016/j.tics.2009.09.003

[ejn70301-bib-0189] Winkler, I. , P. Paavilainen , and R. Näätänen . 1992. “Can Echoic Memory Store two Traces Simultaneously? A Study of Event‐Related Brain Potentials.” Psychophysiology 29, no. 3: 337–349. 1626043 10.1111/j.1469-8986.1992.tb01707.x

[ejn70301-bib-0190] Woolsey, T. A. , and H. Van der Loos . 1970. “The Structural Organization of Layer IV in the Somatosensory Region (SI) of Mouse Cerebral Cortex: The Description of a Cortical Field Composed of Discrete Cytoarchitectonic Units.” Brain Research 17, no. 2: 205–242. 4904874 10.1016/0006-8993(70)90079-x

[ejn70301-bib-0191] Xu, Q. , C. Ye , J. A. Hämäläinen , E. M. Ruohonen , X. Li , and P. Astikainen . 2021. “Magnetoencephalography Responses to Unpredictable and Predictable Rare Somatosensory Stimuli in Healthy Adult Humans.” Frontiers in Human Neuroscience 15: 641273. 33935671 10.3389/fnhum.2021.641273 PMC8079819

[ejn70301-bib-0192] Yabe, H. , M. Tervaniemi , K. Reinikainen , and R. Näätänen . 1997. “Temporal Window of Integration Revealed by MMN to Sound Omission.” Neuroreport 8, no. 8: 1971–1974. 9223087 10.1097/00001756-199705260-00035

[ejn70301-bib-0193] Yamashiro, K. , K. Inui , N. Otsuru , T. Kida , K. Akatsuka , and R. Kakigi . 2008. “Somatosensory off‐Response in Humans: an ERP Study.” Experimental Brain Research 190, no. 2: 207–213. 18584160 10.1007/s00221-008-1468-8

[ejn70301-bib-0194] Yu, A. J. , and P. Dayan . 2005. “Uncertainty, Neuromodulation, and Attention.” Neuron 46, no. 4: 681–692. 15944135 10.1016/j.neuron.2005.04.026

[ejn70301-bib-0195] Yu, X. J. , X. X. Xu , S. He , and J. He . 2009. “Change Detection by Thalamic Reticular Neurons.” Nature Neuroscience 12, no. 9: 1165–1170. 19684591 10.1038/nn.2373

[ejn70301-bib-0196] Zhang, Z. , G. Guo , J. Zhang , et al. 2019. “Do theta Oscillations Explain the Somatosensory Change Detection Mechanism?” Biological Psychology 143: 103–112. 30771407 10.1016/j.biopsycho.2019.02.001

[ejn70301-bib-0197] Zhang, Z. W. , and M. Deschenes . 1998. “Projections to Layer VI of the Posteromedial Barrel Field in the Rat: a Reappraisal of the Role of Corticothalamic Pathways.” Cerebral Cortex 8, no. 5: 428–436. 9722086 10.1093/cercor/8.5.428

